# Supplementary material for: TLR4 endocytosis and endosomal TLR4 signaling are distinct and independent outcomes of TLR4 activation
Source: EMBO Rep. 2025 Apr 9;26(10):2740–66. doi: 10.1038/s44319-025-00444-2 (PMC12116916; doi:10.1038/s44319-025-00444-2)
Supplement: Supplementary file 1 — Appendix [file 44319_2025_444_MOESM1_ESM.pdf]

Appendix for: “TLR4 endocytosis and endosomal TLR4 signaling are distinct and independent outcomes of TLR4 activation”

Appendix Figure S1 2

Appendix Figure S2 4

Appendix Figure S3 6

Appendix Figure S4 8

Appendix Figure S5 10

Appendix Figure S6 12

Appendix Figure S7 14

Appendix Figure S8 16

Appendix Figure S9 18

Appendix Figure S10 20

Appendix Table S1 22

Appendix Table S2 24

Appendix Table S3 26

Appendix Table S4 27

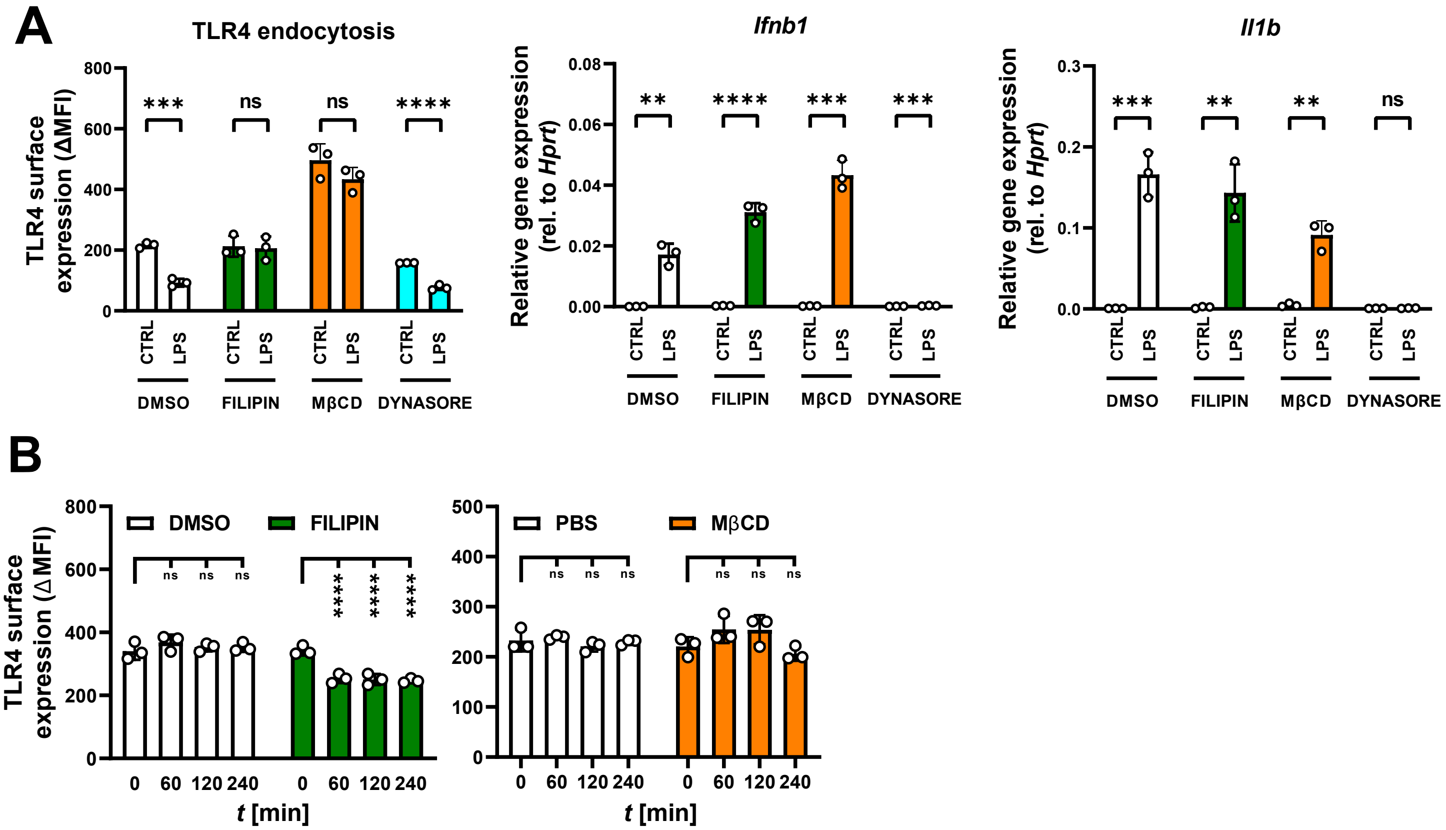

APPENDIX FIGURE S1

Appendix Fig. S1: Related to Figure 1

(A) Flow cytometric analysis of TLR4 surface expression in WT iBMM treated for 60 minutes with DMSO, filipin (5 µM), MβCD (10 mM) or dynasore (80 µM), followed by stimulation with LPS (100 EU/mL) or left unstimulated (CTRL) for 120 minutes. qRT-PCR analysis of *Ifnb1* and *Il1b* expression in WT iBMM treated for 60 minutes with DMSO, filipin (5 µM), MβCD (10 mM) or dynasore (80 µM), followed by stimulation with LPS (100 EU/mL) for 90 minutes. **(B)** Flow cytometric analysis of TLR4 surface expression in WT BMM treated with DMSO, PBS, filipin (5 µM) or MβCD (10 mM) over the indicated time-course. **Data & statistical information.** Bar plots are mean ± S.E.M. of n=3 biological replicates generated in independent experiments indicated as data points. Unpaired two-tailed *t* test performed in A; Ordinary one-way ANOVA with Dunnet's multiple comparisons test performed in B; * P<0.05; **P<0.01; ***P<0.001, ****P<0.0001, ns = not significant.

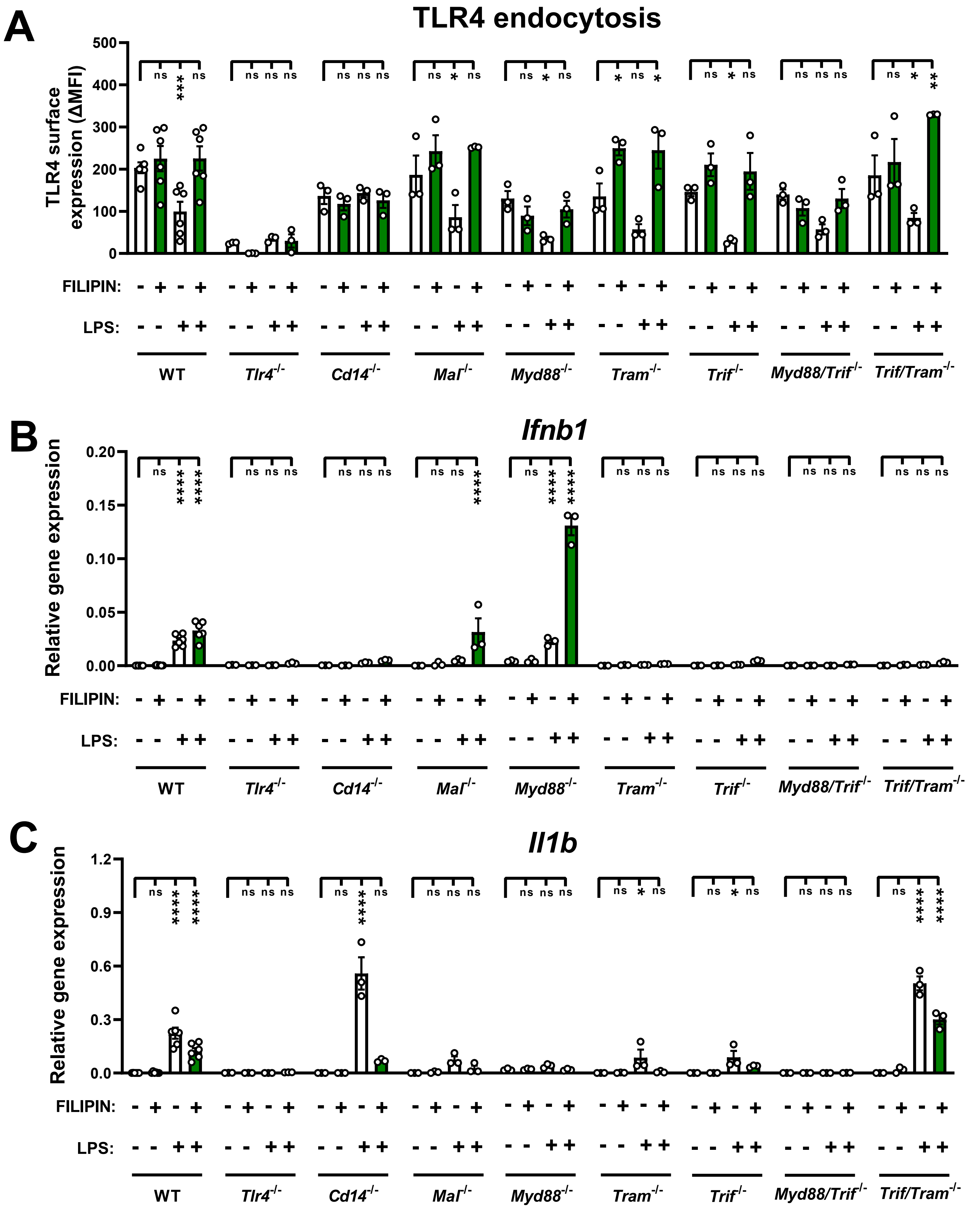

APPENDIX FIGURE S2

Appendix Fig. S2: Related to Figure 1

(A) Flow cytometric analysis of TLR4 endocytosis in iBMM (WT or deficient for the indicated proteins) treated for 60 minutes with filipin (5 µM) or DMSO, followed by stimulation with LPS (100 EU/mL) for 120 minutes. **(B,C)** qRT-PCR analysis of B) *Ifnb1* and C) *Il1b* expression in iBMMs of indicated genotypes treated for 60 minutes with DMSO or filipin (5 µM), followed by stimulation with LPS (100 EU/mL) for 90 minutes. **Data & statistical information.** Bar plots are mean ± S.E.M. of n=3-6 biological replicates generated in independent experiments indicated as data points. Ordinary two-way ANOVA with Dunnet's multiple comparisons test performed in A-C; * P<0.05; **P<0.01; ***P<0.001, ****P<0.0001, ns = not significant.

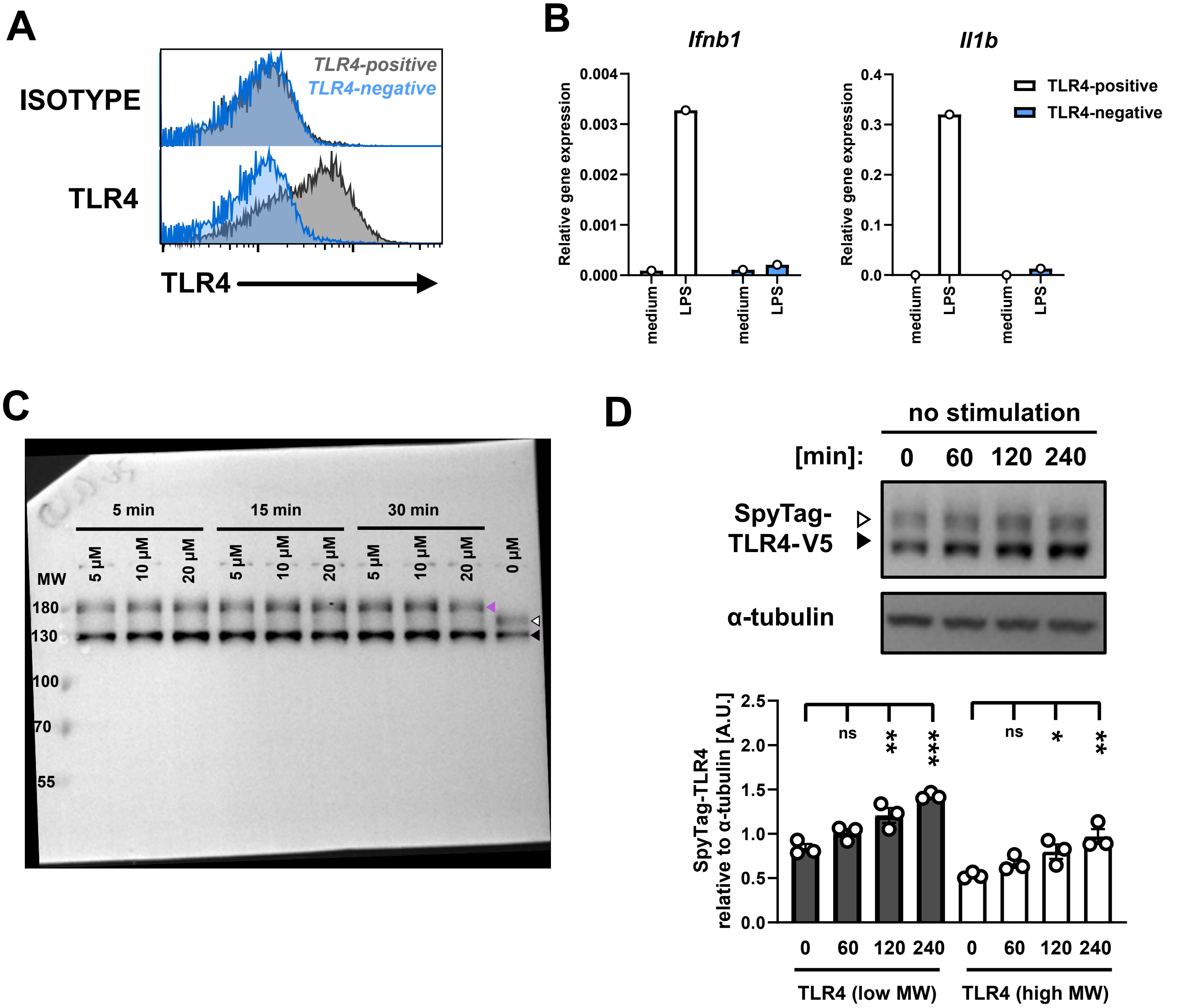

APPENDIX FIGURE S3

Appendix Fig. S3: Related to Figure 1

(A,B) Post-cell sorting flow cytometric analysis of A) TLR4 surface expression and B) LPS-induced *Ifnb1* and *Il1b* expression in RAW^{TLR4ko} cells (TLR4-negative) and TLR4-expressing control population (TLR4-positive). **(C)** Immunoblot analysis of TLR4-V5 in lysates of RAW^{TLR4ko} cells expressing SpyTag-mTLR4-V5. Cells were incubated with SpyCatcher protein (5, 10, or 20 µM) for 0, 15 or 30 minutes. Purple arrow head indicates SpyCatcher-labelled surface TLR4, white and black arrow heads indicate unlabelled cell surface-expressed and intracellular TLR4, respectively. **(D)** Immunoblot analysis of TLR4-V5 in lysates of RAW^{TLR4ko} cells expressing SpyTag-mTLR4-V5. Cells were left unstimulated and cell lysates taken at the indicated intervals. White and black arrow heads indicate unlabelled cell surface-expressed and intracellular TLR4, respectively. **Data & statistical information.** A-C) These data are validation experiments representing a single biological replicate. D) Immunoblot images depict 1 representative of n=3 biological replicates generated in independent experiments. Bar plots are mean ± S.E.M. of n=3 biological replicates generated in independent experiments indicated as data points. Ordinary one-way ANOVA with Dunnett's multiple comparisons test used.

APPENDIX FIGURE S4

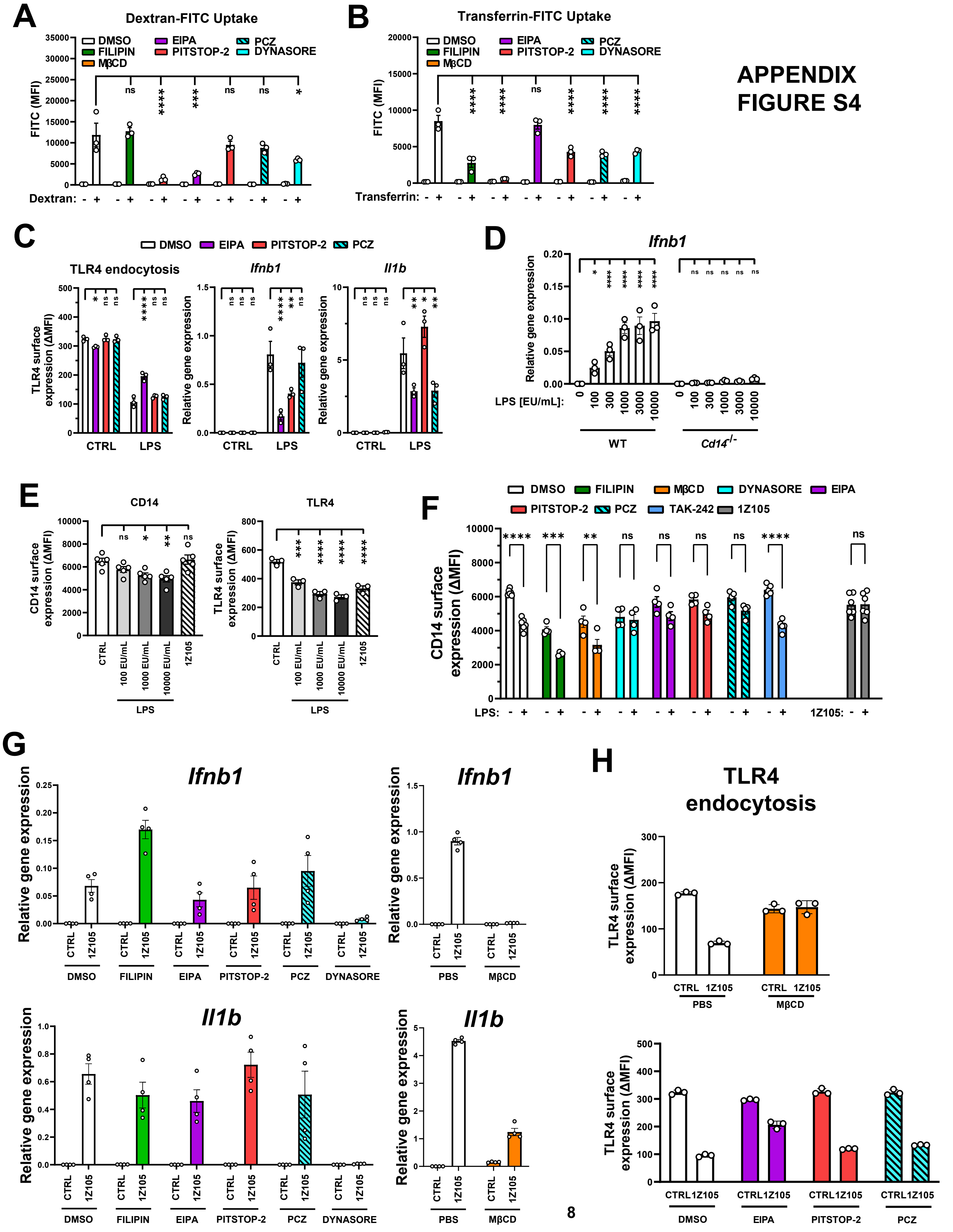

Appendix Fig. S4: Related to Figure 2

(A,B) WT BMM were treated for 60 minutes with DMSO, filipin (5 µM), MβCD (10 mM), EIPA (25 µM), Pitstop-2 (40 µM), PCZ (15 µM), or dynasore (80 µM), followed by incubation with A) dextran-FITC (0.5 mg/mL) or B) transferrin-FITC (12.5 µg/mL) for 120 minutes. Following quenching of surface-bound dextran/transferrin, median fluorescence intensity of intracellular FITC was assessed by flow cytometry. **(C)** Raw data underlying radar plot in Figure 2A. WT BMM were treated for 60 minutes with DMSO, EIPA (25 µM), Pitstop-2 (40 µM) or PCZ (15 µM), followed by LPS stimulation for 90 minutes (*Ifnb1*/*Il1b*) or 120 minutes (TLR4 endocytosis). **(D)** *Ifnb1* expression in WT and *Cd14*$^{-/-}$ iBMM stimulated with the indicated LPS doses for 90 minutes. **(E)** Flow cytometric analysis of CD14 and TLR4 surface expression in WT BMM stimulated with the indicated LPS doses, or 1Z105 (10 µM) for 90 minutes. Data are presented as isotype-subtracted ΔMFI values and normalized to unstimulated control cells in each independent experiment. **(F)** Raw data underlying CD14 endocytosis data in Figure 2A. Flow cytometric analysis of CD14 endocytosis in WT BMM treated for 60 minutes with DMSO or TAK-242 (1 µM), followed by LPS stimulation (10,000 EU/mL) or left untreated for 30 minutes. **(G,H)** Raw data underlying radar plot in Figure 2E. WT BMM were treated for 60 minutes with the indicated inhibitors, followed by 1Z105 stimulation for G) 90 minutes (*Ifnb1*/*Il1b*) or H) 120 minutes (TLR4 endocytosis). **Data & statistical information.** Bar plots are mean ± S.E.M. of n=3-4 biological replicates generated in independent experiments indicated as data points. Ordinary one-way ANOVA with Dunnett's multiple comparisons test in A-E); unpaired two-tailed *t* test in F). * P<0.05; **P<0.01; ***P<0.001, ****P<0.0001, ns = not significant.

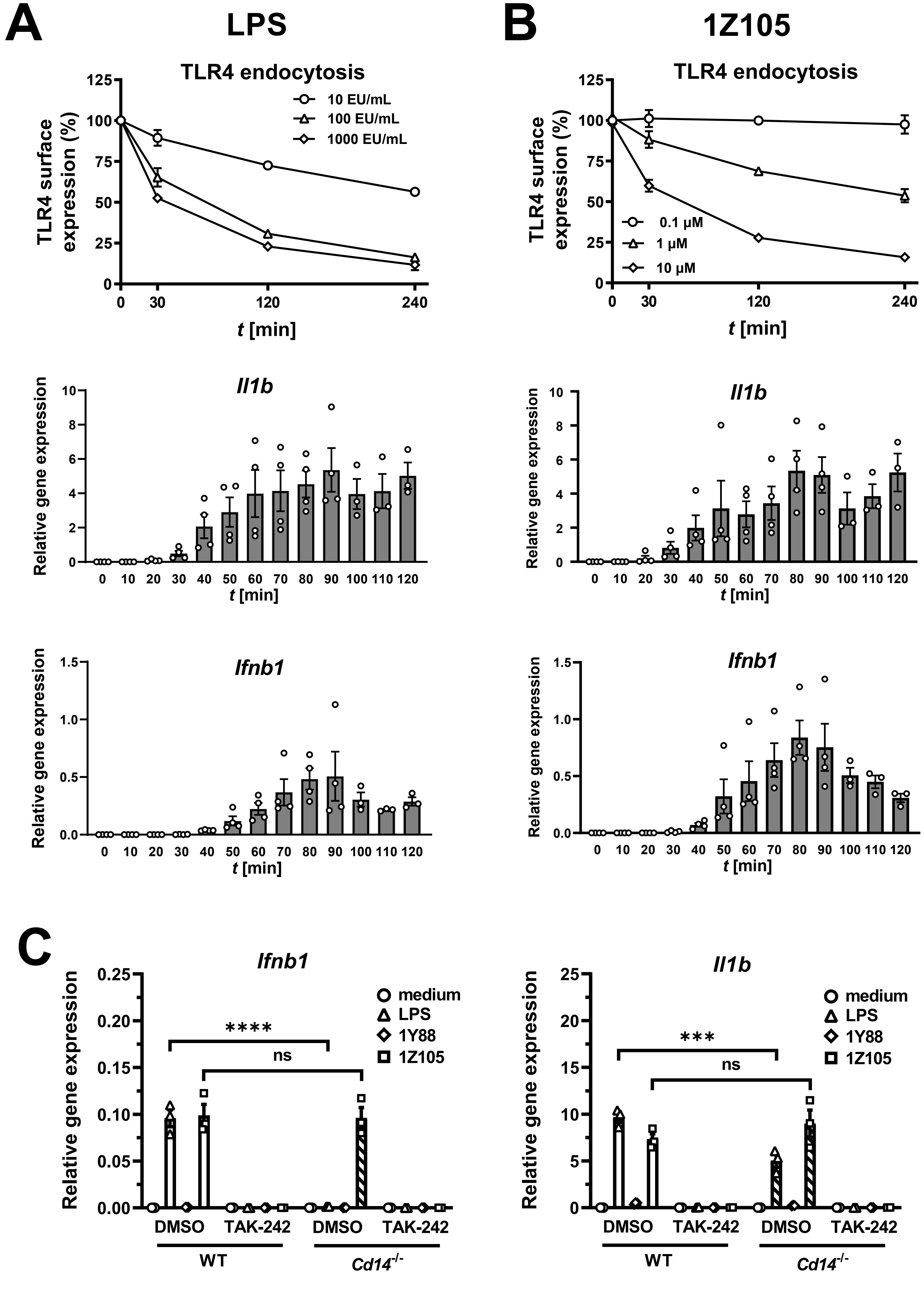

APPENDIX FIGURE S5

Appendix Fig. S5: Related to Figures 3 & 4

(A,B) Time-courses of TLR4 endocytosis, *Ifnb1* expression and *Il1b* expression in WT BMMs stimulated with A) LPS and B) 1Z105 across a dose range. **C)** qRT-PCR analysis of *Ifnb1* and *Il1b* expression in WT and *Cd14*$^{-/-}$ BMMs treated with DMSO or TAK-242 (1 µM) for 60 minutes, then stimulated with LPS (0 or 100 EU/mL), 1Z105 (10 µM) or the inactive control compound, 1Y88 (10 µM) for 90 minutes. **Data & statistical information.** Line graphs are mean ± range of n=2 biological replicates generated in independent experiments indicated as data points. Bar plots are mean ± S.E.M. of n=3-4 biological replicates generated in independent experiments indicated as data points. Ordinary two-way ANOVA with Tukey's multiple comparisons test utilized in C). * P<0.05; **P<0.01; ***P<0.001, ****P<0.0001, ns = not significant.

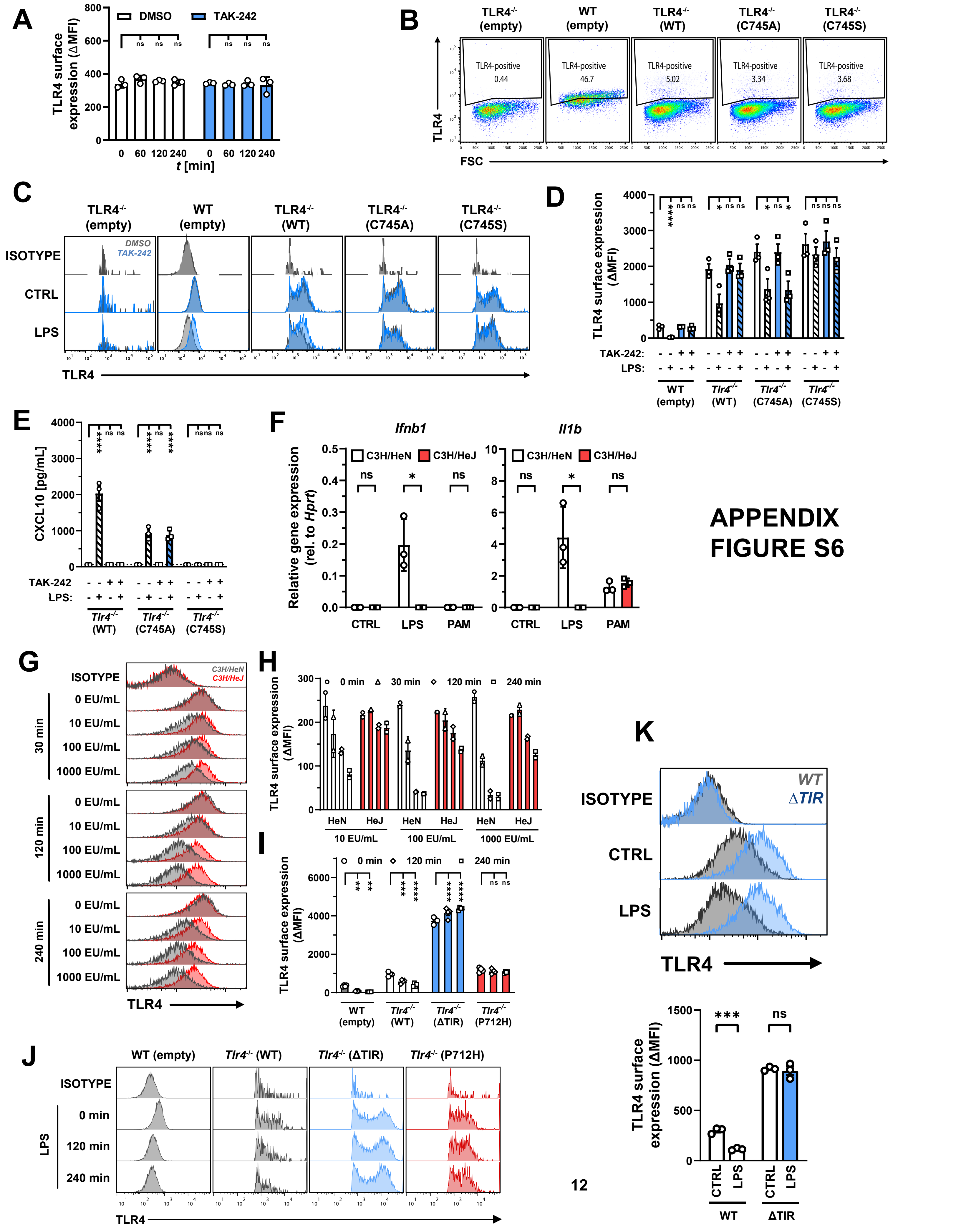

APPENDIX FIGURE S6

Appendix Fig. S6: Related to Figures 3 & 4

(A) TLR4 surface expression in resting WT BMMs exposed to TAK-242 (1 µM) for 0, 60, 120 and 240 minutes. **(B)** Gating strategy for TLR4-expressing cells following retroviral reconstitution of *Tlr4*$^{-/-}$ BMM with mTLR4 constructs. WT transduced with empty vector served as control. Percentages are indicative of proportion of TLR4-positive cells following doublet exclusion. This strategy was used for all retroviral reconstitution experiments. **(C)** Representative histograms of cells detected within the TLR4-positive gate defined in S6B); quantification across independent experiments presented in Figure 3F, S6D. **(D)** MFI values of data presented in Figure 4C prior to normalisation to the respective DMSO-treated/unstimulated sample in each experiment. **(E)** Retroviral reconstitution of *Tlr4*$^{-/-}$ BMMs with mTLR4^{WT}, mTLR4^{C745A} or mTLR4^{C745S} were treated with TAK-242 for 1 hour prior to LPS stimulation (100 EU/mL), with CXCL10 release assessed at 24 hours post-stimulation. (F) *Ifnb1* and *Il1b* mRNA expression (90 minutes) in LPS (100 EU/mL) and Pam_3CSK_4 (10 ng/mL) -stimulated C3H/HeN (wild-type TLR4) and C3H/HeJ (mutant TLR4^{P712H}) BMMs. **(G-J)** G) Representative plots of flow cytometric analysis of TLR4 surface expression in C3H/HeN and C3H/HeJ BMM stimulated with LPS (10, 100 or 1000 EU/mL) over a time-course of 0, 30, 120 and 240 minutes. Quantification in H) shows results from two independent experiments. I) MFI values of data presented in Figure 4D prior to normalisation to relevant unstimulated sample in each independent experiment. J) Representative histograms of events detected within the TLR4-positive gate defined in S6B); quantification across independent experiments presented in Figure 4C, S6I. **(K)** LPS-induced (100 EU/mL) TLR4 endocytosis (120 minutes) in RAW^{TLR4ko} cells stably expressing mTLR4^{WT} and mTLR4$^{\Delta TIR}$. **Data & statistical information.** Flow cytometry histograms and immunoblot images depict 1 representative of n=2-3 biological replicates generated in independent experiments. Bar plots, other than H), are mean ± S.E.M. of n=3 biological replicates generated in independent experiments indicated as data points. For H), bar plots are mean ± range of n=2 biological replicates generated in independent experiments indicated as data points. Ordinary two-way ANOVA with Dunnett's multiple comparisons test utilized in A,I. Ordinary one-way ANOVA with Dunnett's multiple comparisons test utilized in D,E. Unpaired two-tailed *t* test utilized in F,K. * P<0.05; **P<0.01; ***P<0.001, ****P<0.0001, ns = not significant.

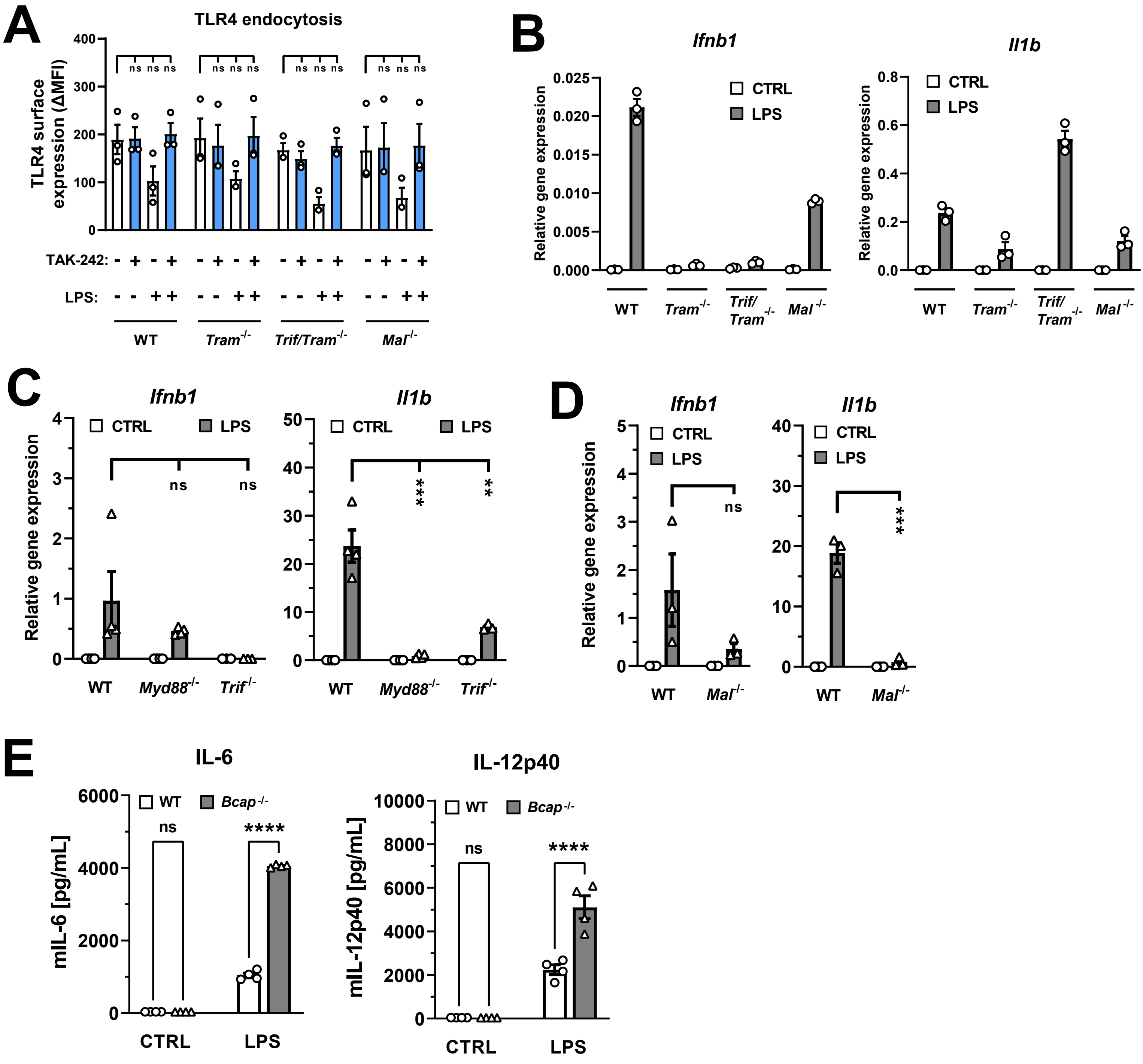

APPENDIX FIGURE S7

Appendix Fig. S7: Related to Figure 5

(A) Flow cytometric analysis of TLR4 surface expression in iBMMs deficient in TLR adaptors, treated with DMSO or TAK-242 (1 µM) for 60 minutes, then stimulated with LPS (0 or 100 EU/mL) for 120 minutes. **(B)** qRT-PCR analysis of *Ifnb1* or *Il1b* expression in iBMMs deficient in TLR adaptors, stimulated with LPS (100 EU/mL), or left unstimulated (CTRL) for 90 minutes. **(C,D)** qRT-PCR analysis of *Ifnb1* or *Il1b* mRNA expression in WT and C) *Myd88*$^{-/-}$, *Trif*$^{-/-}$ and D) *Mal*$^{-/-}$ BMM stimulated with LPS (100 EU/mL) or left unstimulated (CTRL) for 90 minutes. **(E)** ELISA analysis of IL-6 and IL-12p40 in culture supernatant of WT and *Bcap*$^{-/-}$ BMM stimulated with LPS (100 EU/mL) or left unstimulated (CTRL) for 24 hours. **Data & statistical information.** Bar plots are mean ± S.E.M. of n=3-4 biological replicates generated in independent experiments indicated as data points. Ordinary two-way ANOVA with Dunnett's multiple comparisons test utilized in A,C,D. Unpaired two-tailed *t* test utilized in E. * P<0.05; **P<0.01; ***P<0.001, ****P<0.0001, ns = not significant.

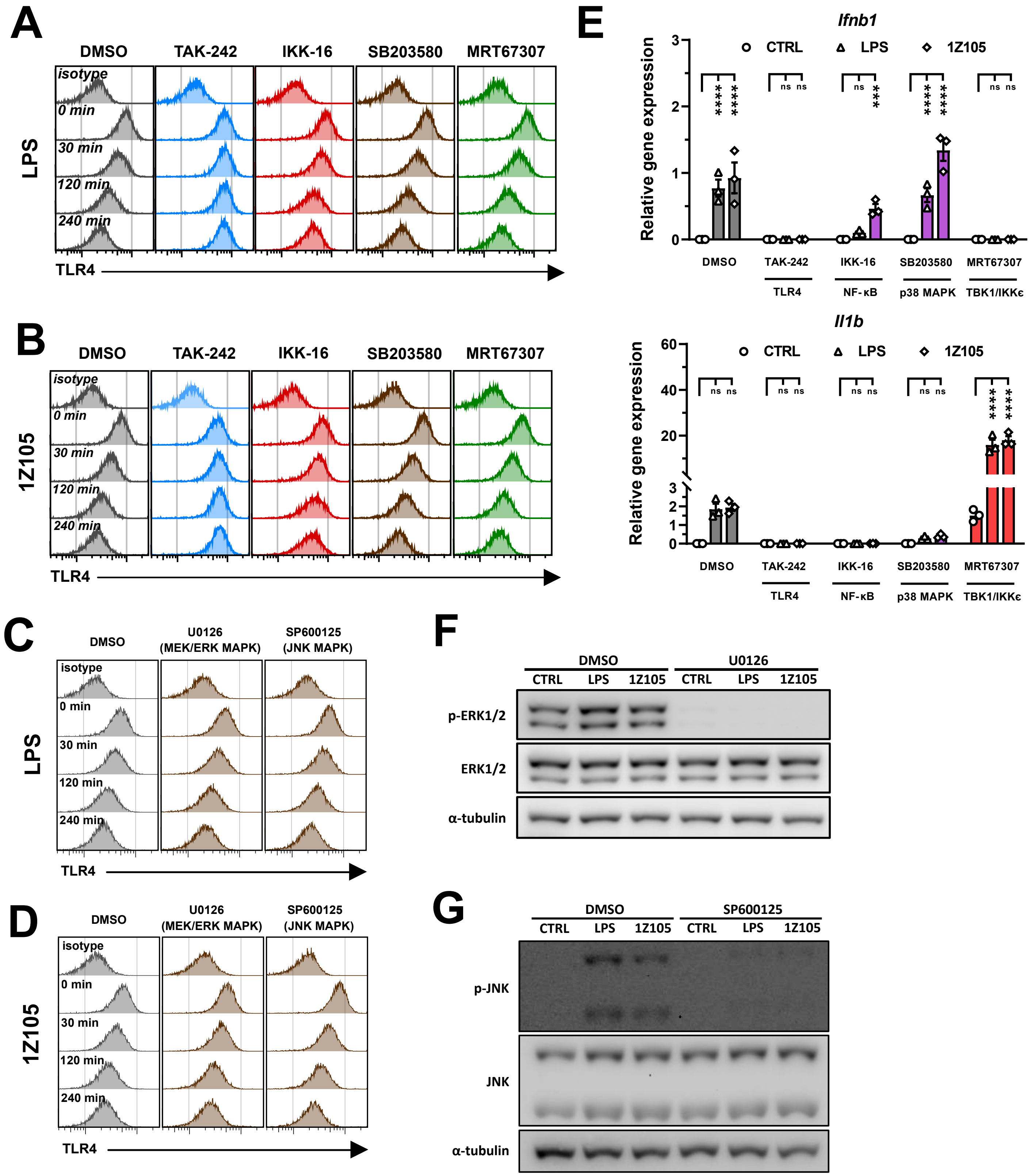

APPENDIX FIGURE S8

Appendix Fig. S8: Relevant to Figure 5

(A,B) Representative flow cytometry histograms of data quantified in Figure 5D, 5E. **(C,D)** Flow cytometric analysis of the impact of canonical TLR signaling pathway (MAPK) inhibitors on TLR4 endocytosis. WT BMM were treated for 60 minutes with DMSO, U0126 (10 µM) or SP600125 (10 µM) followed by stimulation with C) LPS (100 EU/mL) or D) 1Z105 (10 µM) for 0, 30, 120 or 240 minutes. **E)** qRT-PCR analysis of *Ifnb1* and *Il1b* expression in WT BMM treated for 60 minutes with DMSO, TAK-242 (1 µM), IKK-16 (10 µM), SB203580 (10 µM) or MRT67307 (5 µM) followed by stimulation with LPS (100 EU/mL) for 90 minutes. **(F,G)** WT BMM were treated for 60 minutes with DMSO, U0126 (10 µM) or SP600125 (10 µM), followed by stimulation with LPS (100 EU/mL) or 1Z105 (10 µM) for 60 minutes. Phosphorylation of F) ERK1/2 (p42/p44) and G) JNK (p46/p54) were assessed via immunoblot. Sample loading was assessed using total ERK/JNK and alpha-tubulin. Immunoblots are representative of 3 independent experiments. **Data & statistical information.** Flow cytometry histograms and immunoblot images depict 1 representative of n=3 biological replicates generated in independent experiments. Bar plots are mean ± S.E.M. of n=3 biological replicates generated in independent experiments indicated as data points. Ordinary two-way ANOVA with Dunnett's multiple comparisons test was utilized in E. * $P<0.05$; ** $P<0.01$; *** $P<0.001$, **** $P<0.0001$, ns = not significant.

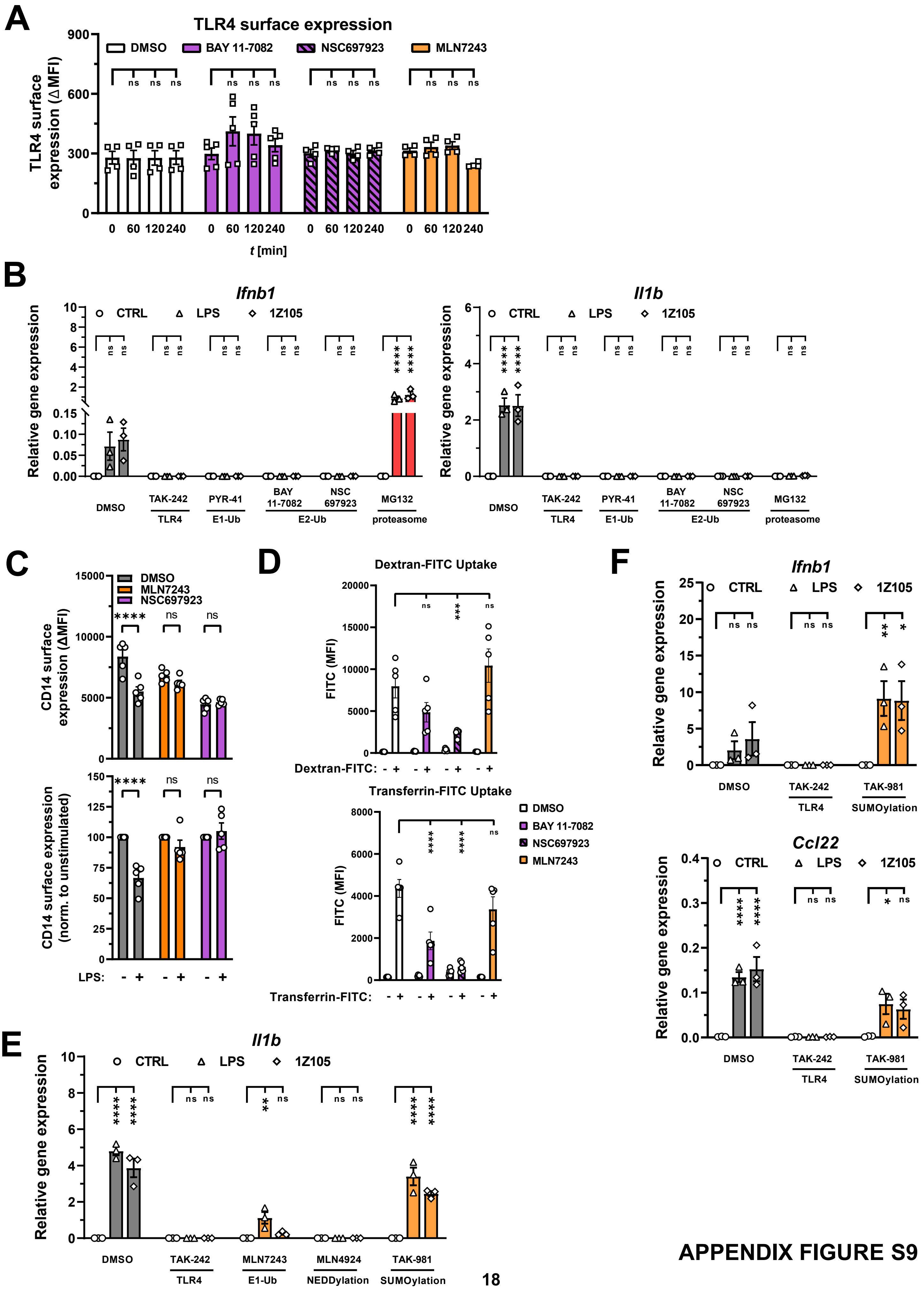

APPENDIX FIGURE S9

Appendix Fig. S9: Related to Figure 6

(A) Flow cytometric analysis of TLR4 surface expression in WT BMM treated with BAY 11-7082 (10 µM), NSC697923 (10 µM) or MLN7243 (1 µM) for 0, 60, 120 or 240 minutes. **(B)** qRT-PCR analysis of *Ifnb1* and *Il1b* expression in WT BMM treated for 60 minutes with DMSO, TAK-242 (1 µM), BAY 11-7082 (10 µM), NSC697923 (10 µM), PYR-41 (25 µM) or MG132 (25 µM), followed by stimulation with LPS (100 EU/mL) or 1Z105 (10 µM) for 90 minutes. **(C)** Flow cytometric analysis of CD14 surface expression in WT BMM treated with DMSO, NSC697923 (10 µM) or MLN7243 (1 µM) for 60 minutes, followed by LPS stimulation (10000 EU/mL) for 30 minutes. **(D)** WT BMM were treated for 60 minutes with DMSO, BAY 11-7082 (10 µM), NSC697923 (10 µM), or MLN7243 (1 µM), followed by incubation with dextran-FITC (0.5 mg/mL) or transferrin-FITC (12.5 µg/mL) for 120 minutes. Following quenching of surface-bound dextran/transferrin with acidic buffer, fluorescence of intracellular FITC was assessed via flow cytometry. **(E,F)** qRT-PCR analysis of E) *Il1b* or F) *Ifnb1* or *Ccl22* expression in WT BMM treated for 60 minutes with DMSO, TAK-242 (1 µM), MLN7243 (1 µM), MLN4924 (2.5 µM) or TAK-981 (2 µM) followed by stimulation with LPS (100 EU/mL) or 1Z105 (10 µM) for 90 minutes. **Data & statistical information.** Bar plots are mean ± S.E.M. of n=3-5 biological replicates generated in independent experiments indicated as data points. Ordinary two-way ANOVA with Sidak's multiple comparisons test was utilized in A; ordinary two-way ANOVA with Dunnett's multiple comparisons test was utilized in B; unpaired two-tailed t test utilized in was utilized in C; ordinary two-way ANOVA with Sidak's multiple comparisons test was utilized in D; ordinary two-way ANOVA with Tukey's multiple comparisons test was utilized in E,F); *P<0.05; **P<0.01; ***P<0.001, ****P<0.0001, ns = not significant. Data presented as mean ± S.E.M.

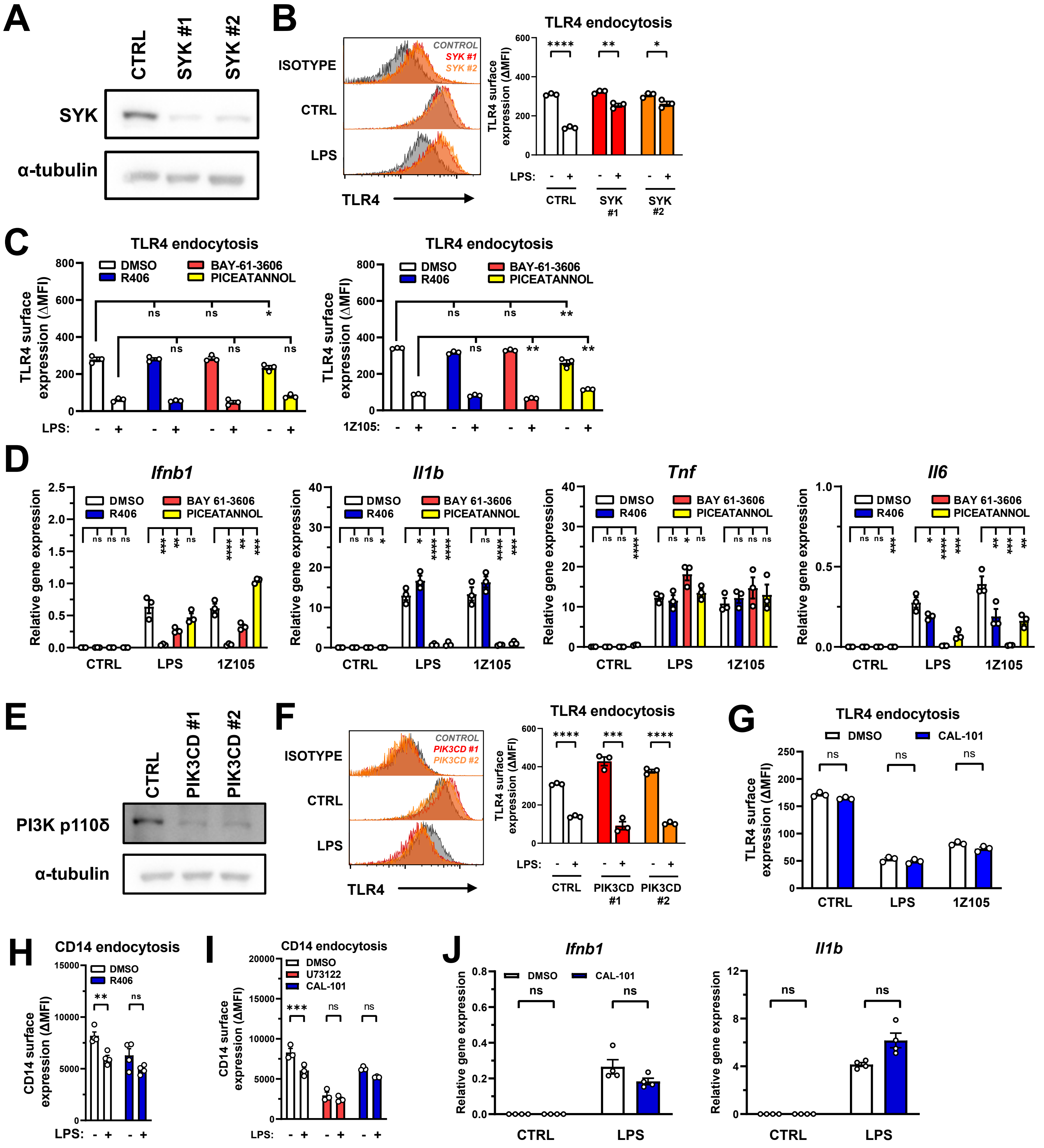

APPENDIX FIGURE S10

Appendix Fig. S10: Related to Figure 6 and 7

(A,B) CRISPR/Cas9-mediated knockdown of SYK expression in WT BMM. A) SYK knockdown was verified by immunoblot analysis, and B) LPS-induced (100 EU/mL, 120 minutes) TLR4 endocytosis assessed via flow cytometry. **(C)** WT BMM were treated for 60 minutes with DMSO, R406 (10 µM), BAY 61-3606 (10 µM or) piceatannol (75 µM), followed by stimulation with LPS (100 EU/mL) or 1Z105 (10 µM) for 120 minutes. TLR4 surface expression was assessed via flow cytometry. **(D)** qRT-PCR analysis of *Ifnb1*, *Il1b*, *Tnf* and *Il6* expression in WT BMM treated for 60 minutes with DMSO, R406 (10 µM), BAY 61-3606 (10 µM) or piceatannol (75 µM), followed by stimulation with LPS (100 EU/mL) or 1Z105 (10 µM) for 90 minutes. **(E,F)** CRISPR/Cas9-mediated knockdown of PI3K p110δ expression in WT BMM. E) PI3K p110δ knockdown was verified by immunoblot analysis, and F) LPS-induced (100 EU/mL, 120 minutes) TLR4 endocytosis assessed via flow cytometry. **(G)** Flow cytometric analysis of TLR4 surface expression in WT BMM treated with CAL-101 (10 µM) for 60 minutes, followed by LPS (100 EU/mL) or 1Z105 (10 µM) stimulation for 120 minutes. **(H)** Flow cytometric analysis of CD14 endocytosis in WT BMM treated with DMSO or R406 (10 µM) for 60 minutes, followed by LPS stimulation (10,000 EU/mL) for 30 minutes. **(I)** Flow cytometric analysis of CD14 endocytosis in WT BMM treated with DMSO, U73122 (10 µM) or CAL-101 (10 µM) for 60 minutes, followed by LPS stimulation (10,000 EU/mL) for 30 minutes. **(J)** qRT-PCR analysis of *Ifnb1* and *Il1b* expression in WT BMM treated for 60 minutes with CAL-101 (10 µM), followed by stimulation with LPS (100 EU/mL) for 90 minutes. **Data & statistical information.** Flow cytometry histograms and immunoblot images depict 1 representative of n=3 biological replicates generated in independent experiments. Bar plots are mean ± S.E.M. of n=3-4 biological replicates generated in independent experiments indicated as data points. Unpaired two-tailed t test utilized in B,F-J. Ordinary one-way ANOVA with Dunnett's multiple comparisons test utilized in C,D. * $P<0.05$; ** $P<0.01$; *** $P<0.001$, **** $P<0.0001$, ns = not significant. Unstimulated DMSO-treated samples in H) are shared with I).

<u>Appendix Table S1:</u> Details of small molecule inhibitors used in this study

Inhibitor	Supplier	SKU	Working concentration	Solvent
TAK-242 (CLI-095)	InvivoGen	Cat# tlrl-cli95	1 µM	DMSO
Filipin III from *Streptomyces filipinensis*	Sigma-Aldrich	Cat# F4767	5 µM	DMSO
Methyl-β-cyclodextrin	Sigma-Aldrich	Cat# C4555	10 mM	PBS
Dynasore hydrate	Sigma-Aldrich	Cat# 7693	80 µM	DMSO
EIPA	Cayman Chemicals	Cat# 14406	25 µM	DMSO
Pitstop-2	Sigma-Aldrich	Cat# SML1169	40 µM	DMSO
Prochlorperazine	Sigma-Aldrich	Cat# P9178	15 µM	DMSO
BAY 11-7082	Selleck Chemicals	Cat# S2913	10 µM	DMSO
IKK-16	Selleck Chemicals	Cat# S2882	10 µM	DMSO
MRT67307	Selleck Chemicals	Cat# S1102	5 µM	DMSO
U0126	Selleck Chemicals	Cat# S1460	10 µM	DMSO
SP600125	Selleck Chemicals	Cat# S1076	10 µM	DMSO
SB203580	Selleck Chemicals	Cat# S7948	10 µM	DMSO
PYR-41	Selleck Chemicals	Cat# S7129	25 µM	DMSO
NSC697923	Selleck Chemicals	Cat# S7142	10 µM	DMSO
MG132	Selleck Chemicals	Cat #S2619	25 µM	DMSO
TAK-981	Cayman Chemicals	Cat# 32741	1 µM	DMSO
MLN4924	Cayman Chemicals	Cat# 15217	2.5µM	DMSO

MLN7243	Cayman Chemicals	Cat# 30108	1 µM	DMSO
U73122	Selleck Chemicals	Cat# S8011	10 µM	DMSO
CAL-101	Selleck Chemicals	Cat# S2226	10 µM	DMSO
R406	Selleck Chemicals	Cat# S1533	10 µM	DMSO
BAY 61-3606	Selleck Chemicals	Cat# S7006	10 µM	DMSO
Piceatannol	Tocris	Cat# 1554	75 µM	DMSO
SC-514	Enzo Life Sciences	Cat# BML-EI343	20 µM	DMSO

Appendix Table S2: Site-directed mutagenesis primers used in this study

Purpose	Primer	Sequence
Deletion of mTLR4 sequence to express only linker-V5-HIS from WT backbone	FWD	AAGGGCAATTCTGCAGATATC
	REV	CATGGTGAAGCCTGCTTT
mTLR4-C745A mutant from WT backbone	FWD	GAGCCGTTGGGCTATCTTTGAATATGAG
	REV	TGAATAAAGTGTCTAGACAC
mTLR4-C745S mutant from WT backbone	FWD	GAGCCGTTGGTCTATCTTTGAATATG
	REV	TGAATAAAGTGTCTAGACAC
mTLR4-P712H mutant from WT backbone	FWD	AGACTTTATTCACGGTGTAGCCATTG
	REV	CTGTAGTGAAGGCAGAGG
mTLR4-ΔTIR(659) mutant from WT backbone	FWD	AAGGGCAATTCTGCAGATATC
	REV	AATAAGTATCAGGTGAAAATAGAAG
mTLR4-ΔTIR(650) mutant from WT backbone	FWD	AAGGGCAATTCTGCAGATATC
	REV	GTATATCAGAAATGCTACAGTG
mTLR4-ΔTIR(670) mutant from WT backbone	FWD	AAGGGCAATTCTGCAGATATC
	REV	GCTTTCTCCTCTGCTGTAC
mTLR4-K663R mutant from WT backbone	FWD	TGCTGGCTGTAGAAAGTACAGCA
	REV	ATAAGTATCAGGTGAAAATAGAAGTG
mTLR4-K663/664R mutant from K663R backbone	FWD	TGGCTGTAGAAGGTACAGCAGAG
	REV	GCAATAAGTATCAGGTGAAAATAG
mTLR4-K692R mutant from WT backbone	FWD	TGAGCTGGTAAGGAATTTAGAAGAAG
	REV	TTTCTCACCCAGTCCTCATTC

mTLR4-K727R mutant from WT backbone	FWD	AGGCTTCCACAGGAGCCGGAAGG
	REV	TCCTGGATGATGTTGGCAGC
mTLR4-K730R mutant from K727R backbone	FWD	CAGGAGCCGGAGGGTTATTGTGG
	REV	TGGAAGCCTTCCTGGATG
mTLR4-K771R mutant from WT backbone	FWD	TGTCCTTGAGCGGGTTGAGAAGTC
	REV	ATGAAGATGATGCCAGAG
mTLR4-K771/774R mutant from K771R backbone	FWD	GCGGGTTGAGAGGTCCCTGCTGA
	REV	TCAAGGACAATGAAGATGATGCCAGAG
mTLR4-K810R mutant from WT backbone	FWD	GAGAAGACTTAGAAATGCCCTATTGG
	REV	CAGAAGATGTGCCTCCCC
mTLR4-K817R mutant from WT backbone	FWD	ATTGGATGGACGGGCCTCGAATCCTG
	REV	AGGGCATTTTTAAGTCTTC
SpyTag003 insertion into pEF-mTLR4-V5-HIS	FWD	GGTGGACGCCTACAAACGCTATAAAAATCCCTGCATAGAGGTAG
	REV	ATAACAATATGAGGAACGCCACGTCCCAAGCTTCCTGGTGTCAG

<u>Appendix Table S3:</u> qRT-PCR primers used in this study

**Primer**	**Sequence**
m*Hprt*-FW	CCCCAAAATGGTTAAGGTTGC
m*Hprt*-RV	AACAAAGTCTGGCCTGTATCC
m*Tnf*-FW	CATCTTCTCAAAATTCGAGTGACAA
m*Tnf*-RV	TGGGAGTAGACAAGGTACAACCC
m*Il6*-FW	GAGGATACCACTCCCAACAGACC
m*Il6*-RV	AAGTGCATCATCGTTGTTCATACA
m*Il1b*-FW	GTTGATTCAAGGGGACATTA
m*Il1b*-RV	AGCTTCAATGAAAGACCTCA
m*Ifnb1*-FW	CGAGCAGAGATCTTCAGGAAC
m*Ifnb1*-RV	TCCGCCTCTGATGCTTAAAG
m*Ccl22*-FW	TCCCTATGGTGCCAATGTG
m*Ccl22*-RV	ATATCTCGGTTCTTGACGGTTATC

<u>Appendix Table S4:</u> CRISPR/Cas9 crRNA guides used in this study

**Primer**	**Sequence**
mSyk #1	GCCATTAAGTTCCCTCTCGA
mSyk #2	ATTGCACTACCGCATTGACA
mPlcg2 #1	AGATAAAGGAAATCCGTCCG
mPlcg2 #2	GGTGTCCACGTTGACCATGG
mPik3cd #1	CTGGTCAACGTGAAGTTCGA
mPik3cd #2	CACCTGTGTGAACCAGACGG
mTlr4	TAATATTACCTACCAATGCA
