# Supplementary figures and images for: TLR4 endocytosis and endosomal TLR4 signaling are distinct and independent outcomes of TLR4 activation
Source: EMBO Rep. 2025 Apr 9;26(10):2740–66. doi: 10.1038/s44319-025-00444-2 (PMC12116916; doi:10.1038/s44319-025-00444-2)

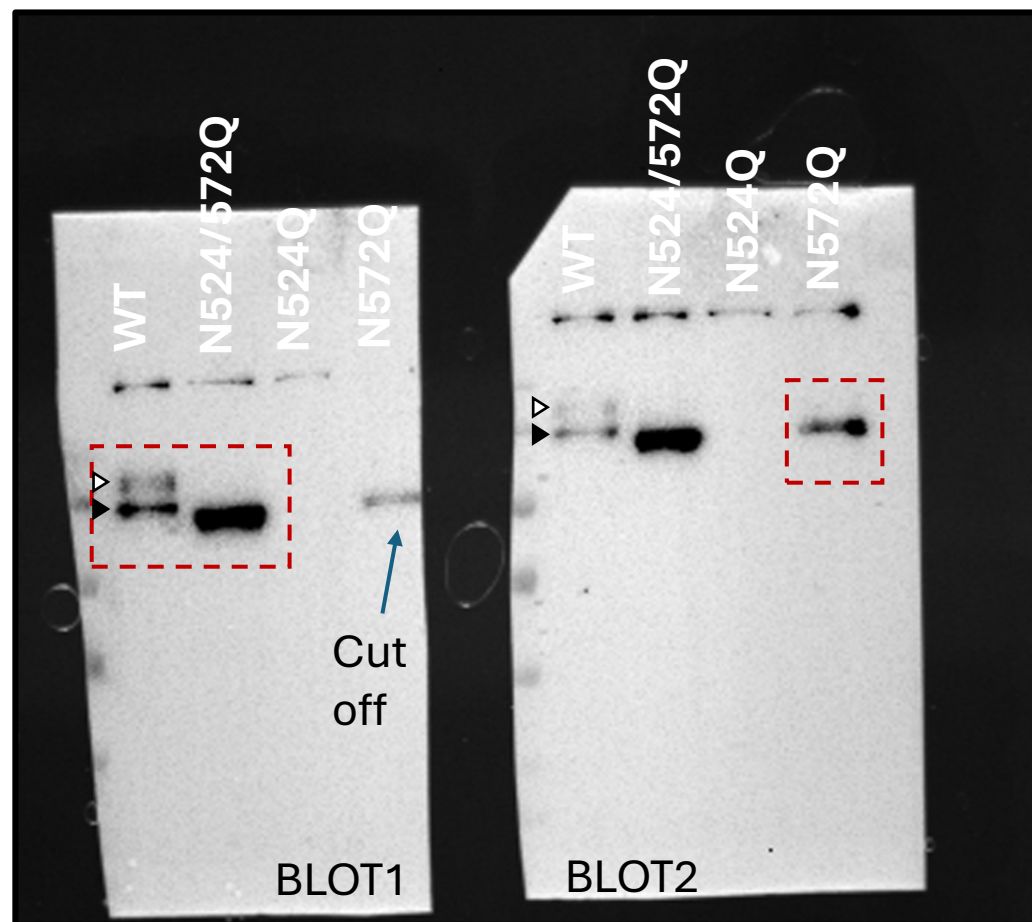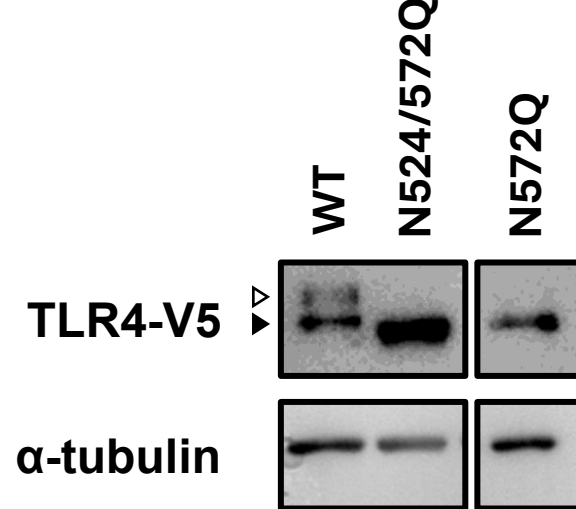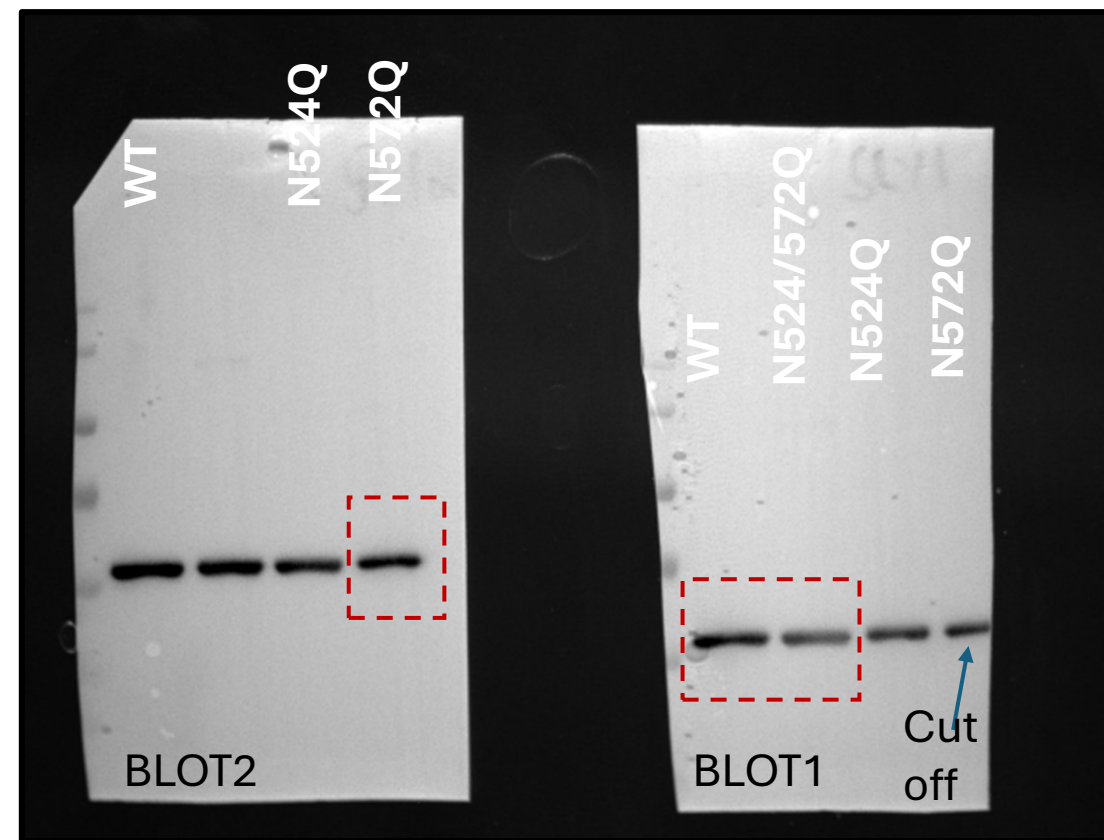

Supplement: Supplementary file 3 — Source data Fig. 1 [file 44319_2025_444_MOESM3_ESM.zip › Figure 1/1E/1E - full blot images.pdf]

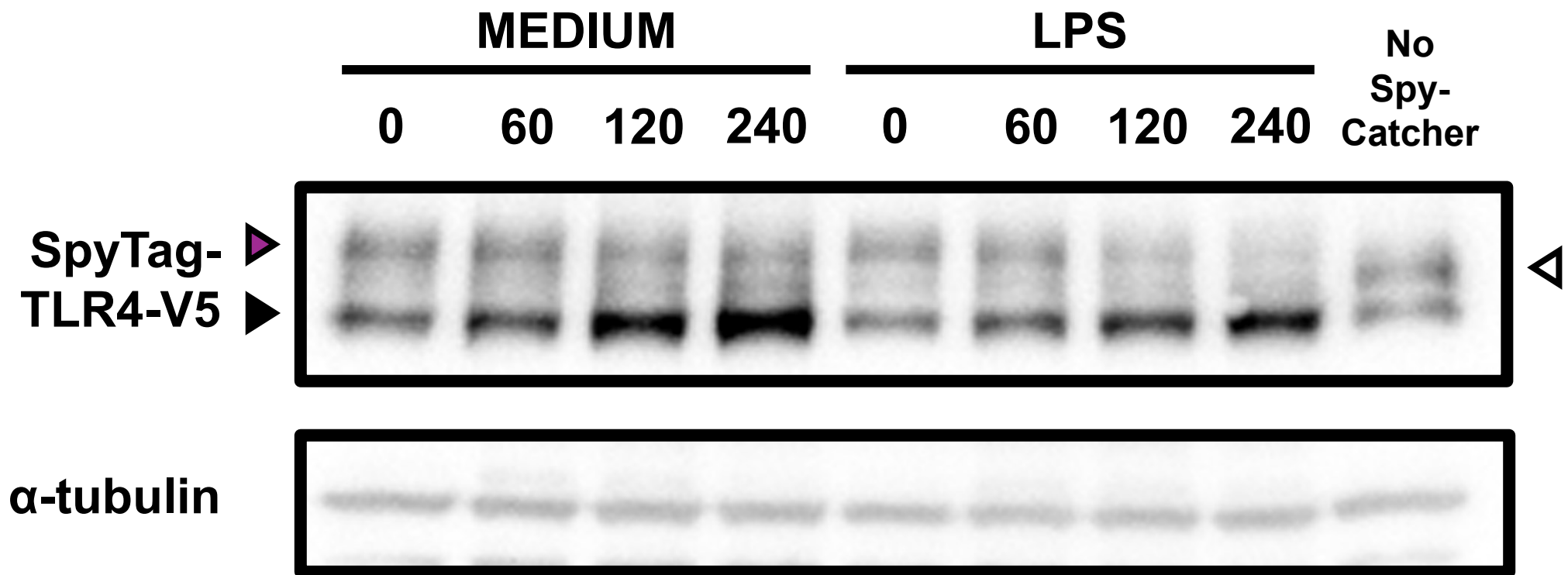

SpyCatcher-  
TLR4-V5

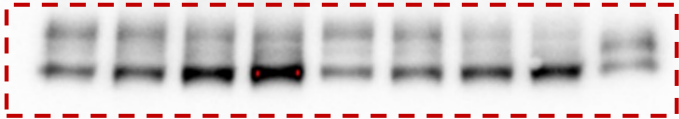

a-tubulin

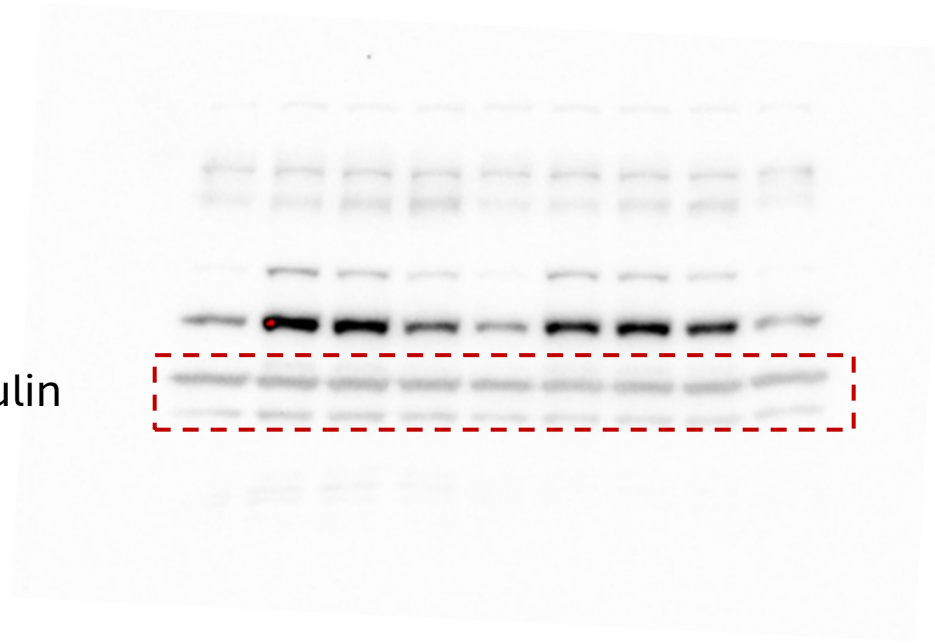

Supplement: Supplementary file 3 — Source data Fig. 1 [file 44319_2025_444_MOESM3_ESM.zip › Figure 1/1H/1H - full blot images.pdf]

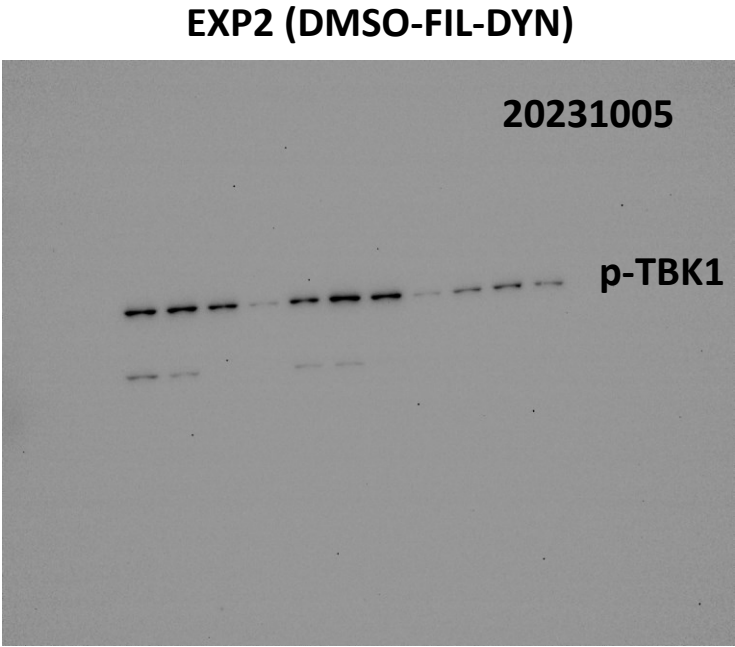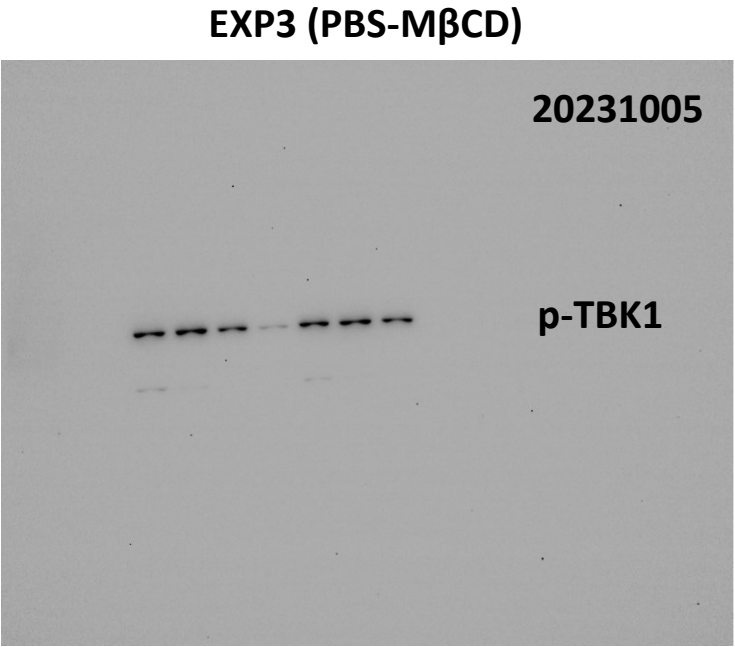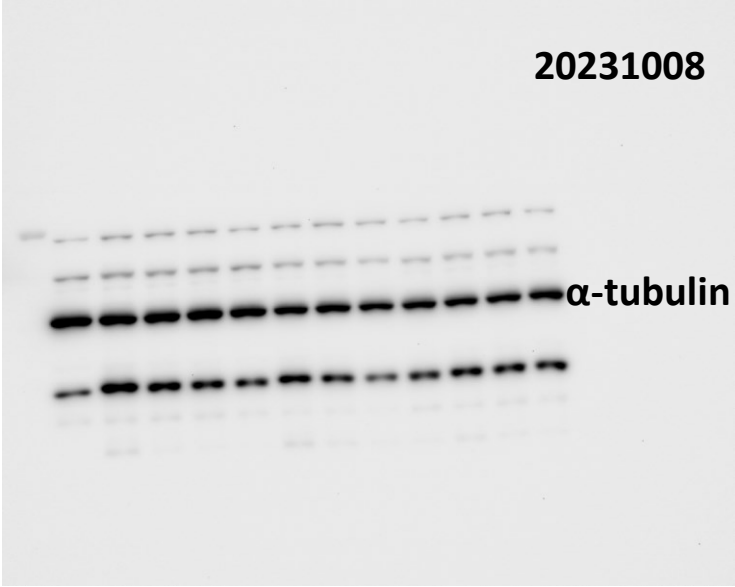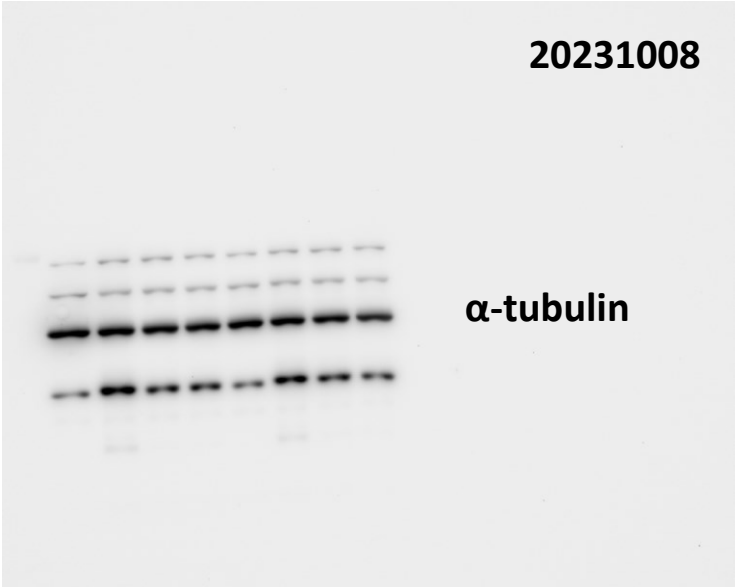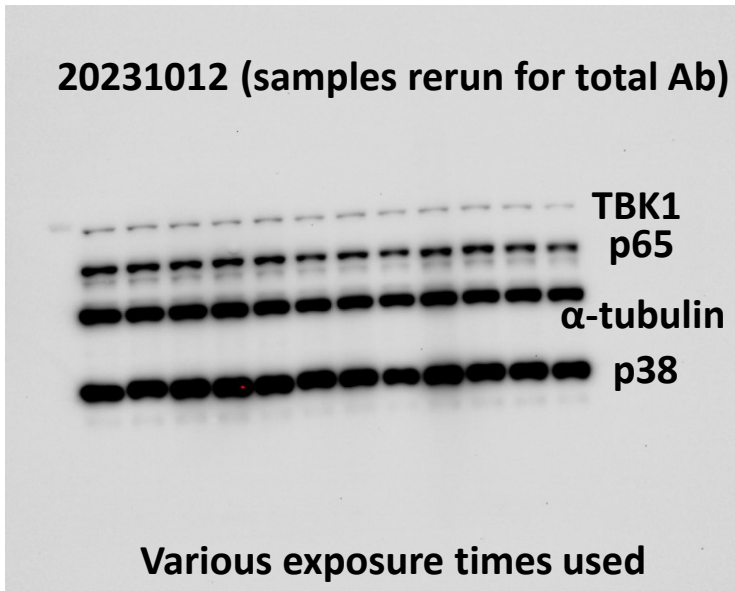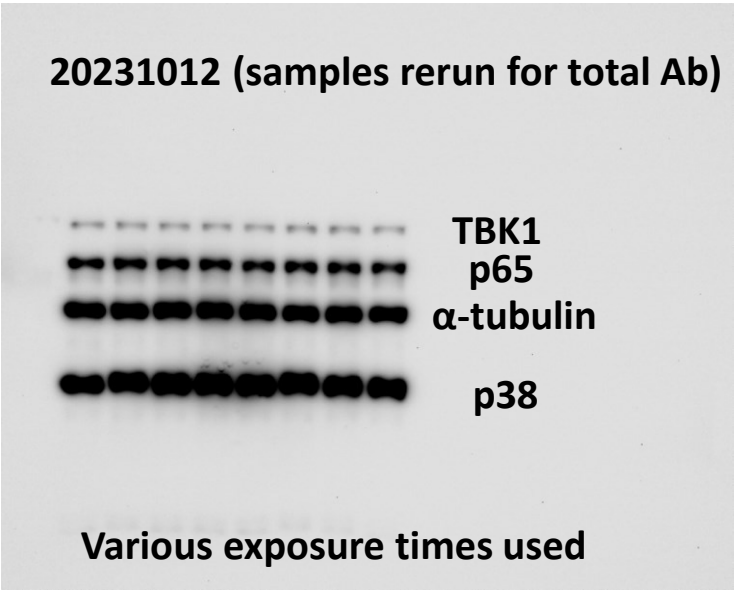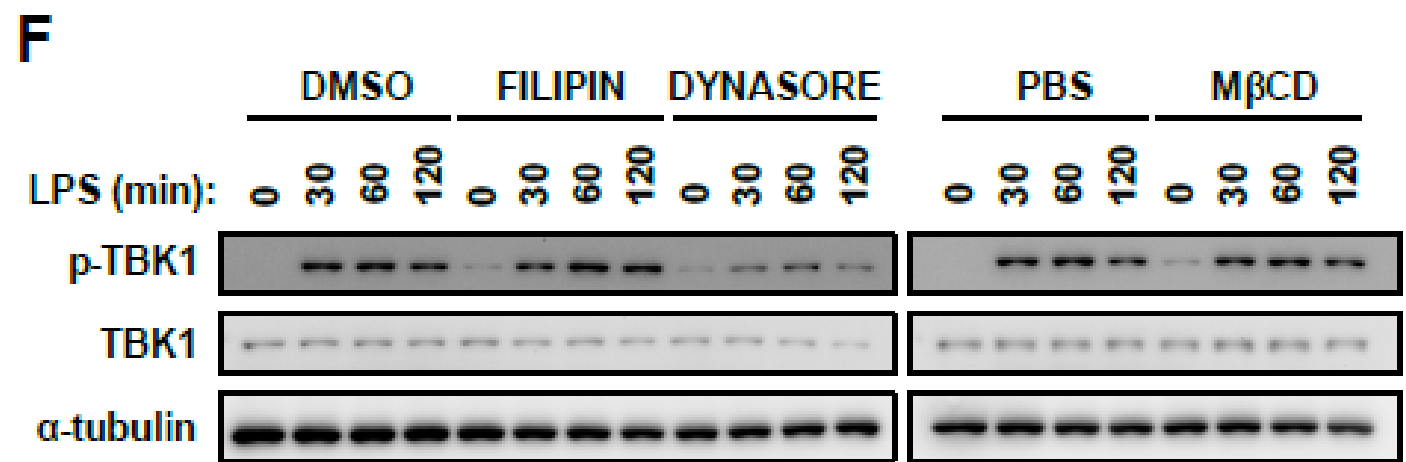

Supplement: Supplementary file 10 — EV Figures Source Data [file 44319_2025_444_MOESM10_ESM.zip › EV Figures/Figure EV1/EV1F/EV1F - full blot images.pdf]

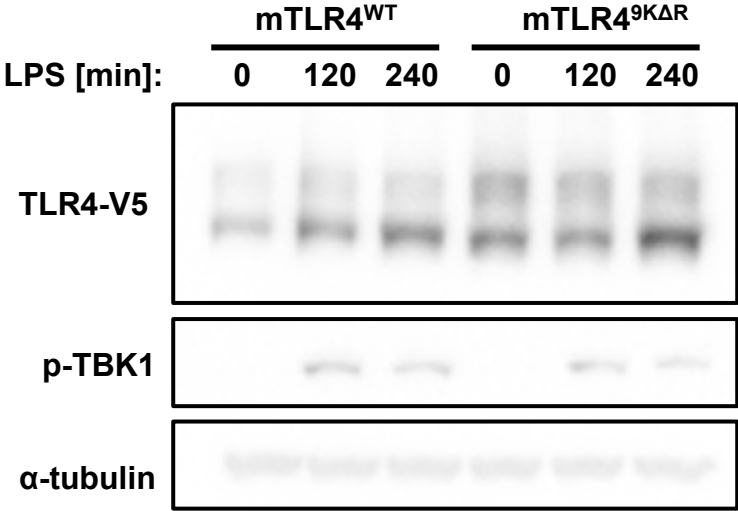

TLR4-V5

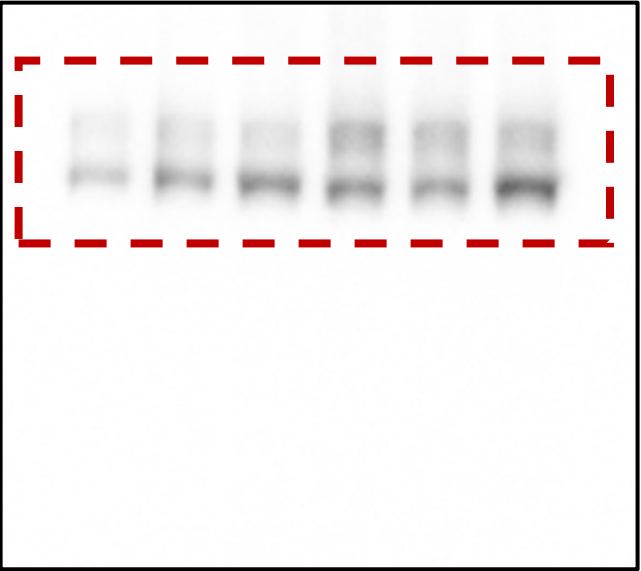

p-TBK1

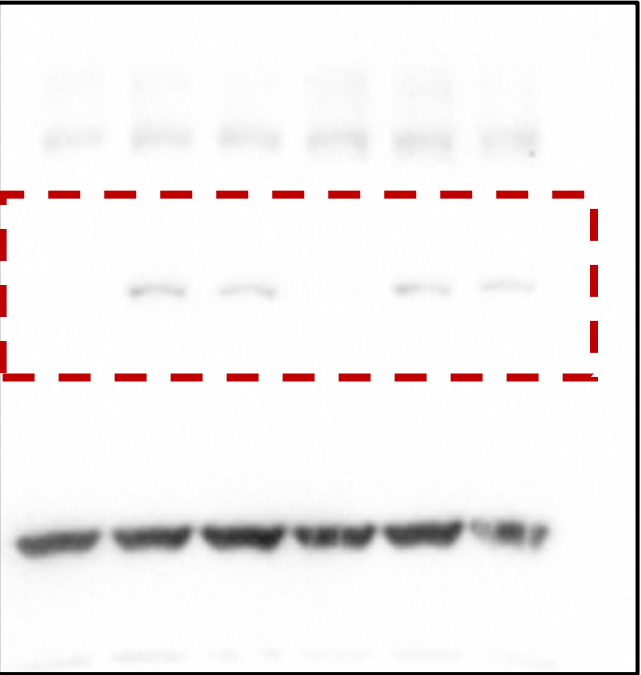

α-tubulin

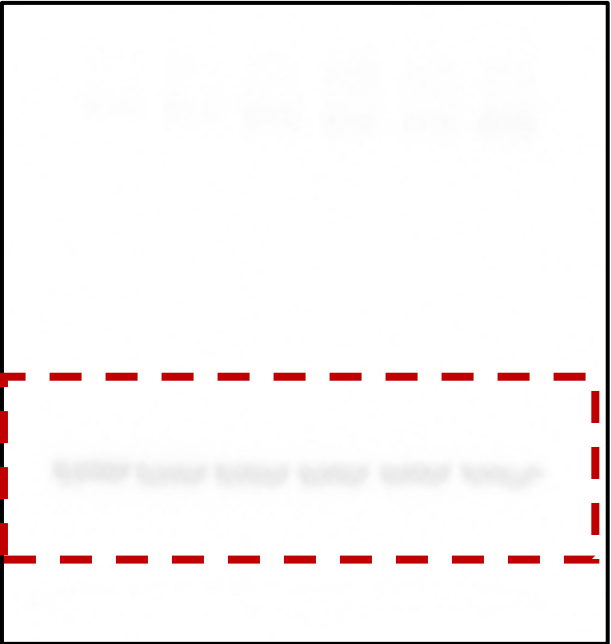

Supplement: Supplementary file 10 — EV Figures Source Data [file 44319_2025_444_MOESM10_ESM.zip › EV Figures/Figure EV2/EV2D/EV2D - full blot images.pdf]

**EV3B**

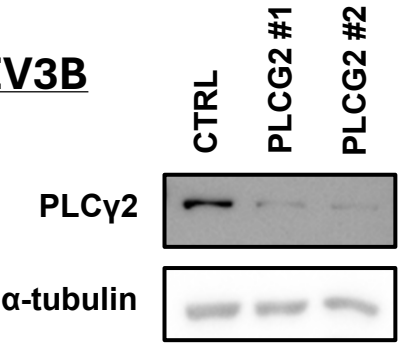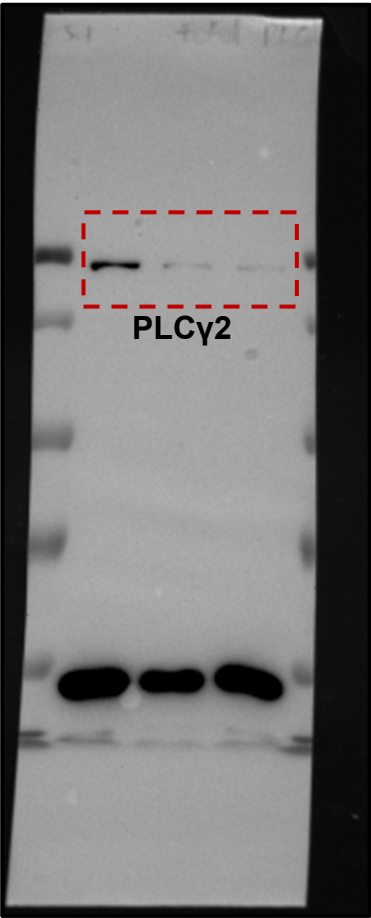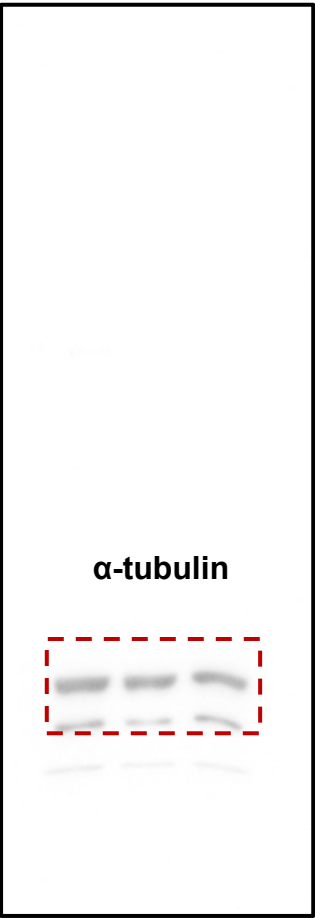

**S10A**

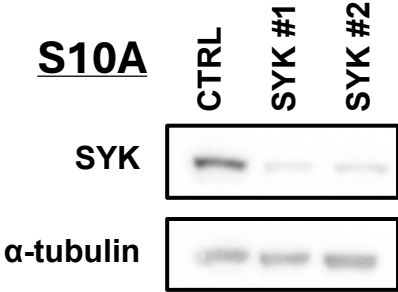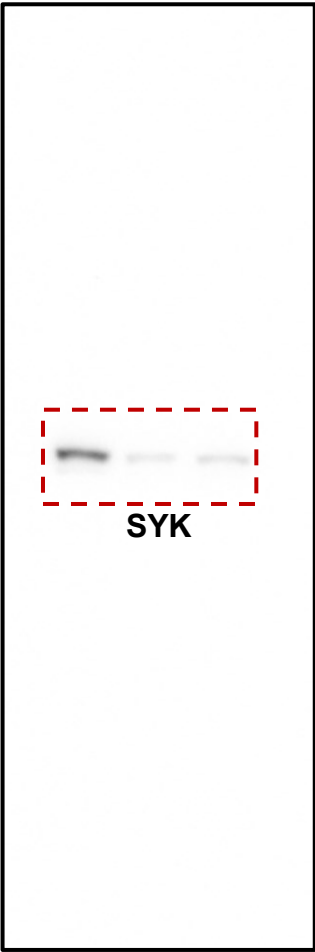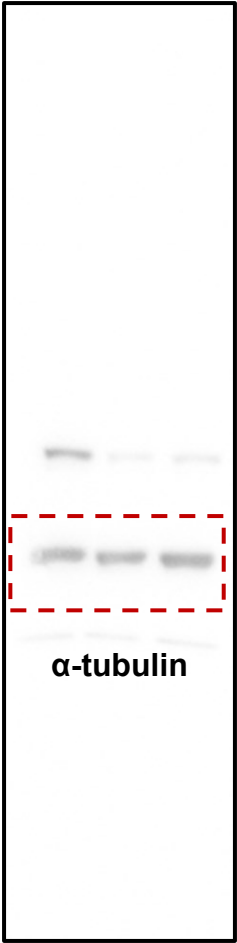

**S10E**

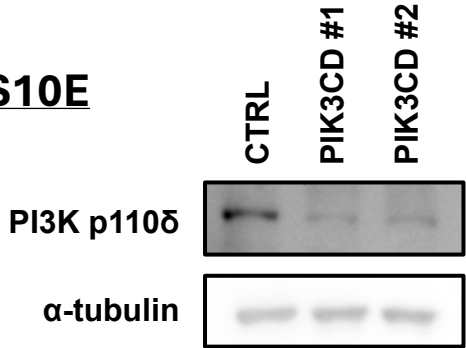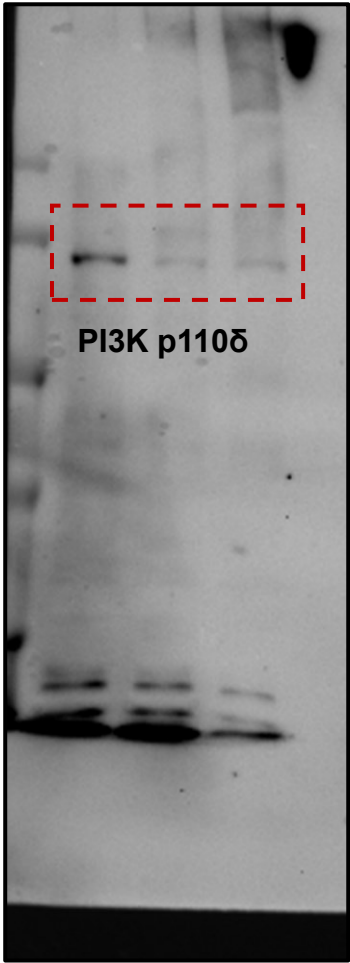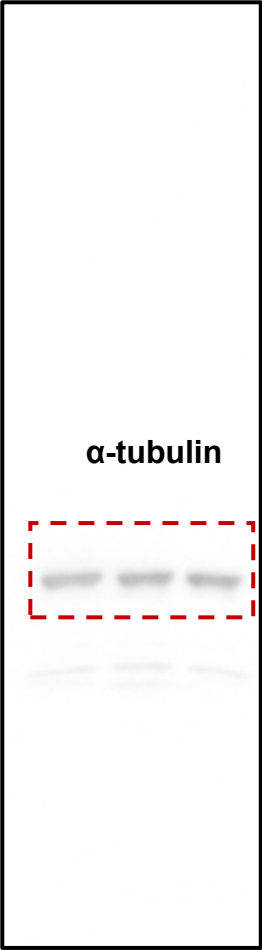

Supplement: Supplementary file 10 — EV Figures Source Data [file 44319_2025_444_MOESM10_ESM.zip › EV Figures/Figure EV3/EV3B/EV3B - full blot images.pdf]

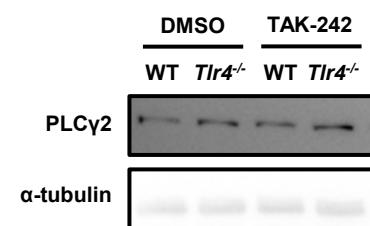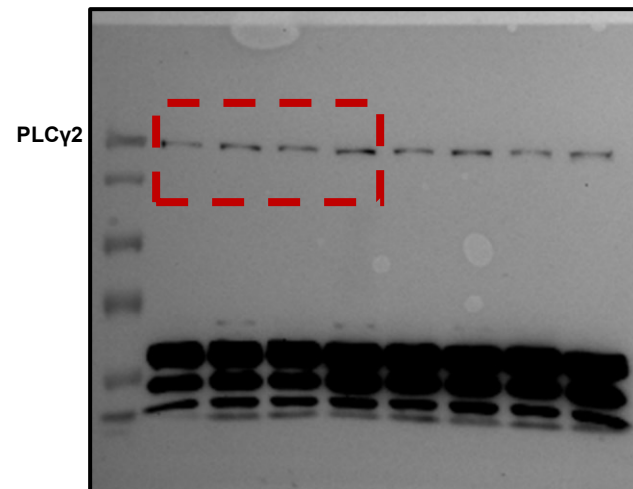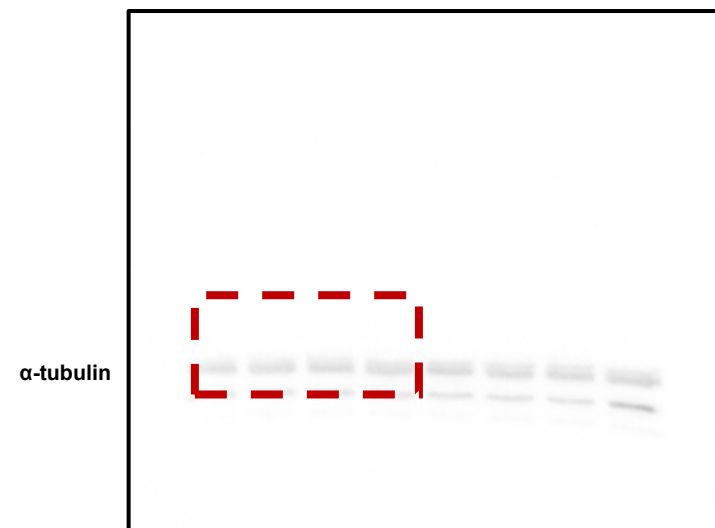

Supplement: Supplementary file 10 — EV Figures Source Data [file 44319_2025_444_MOESM10_ESM.zip › EV Figures/Figure EV3/EV3H/EV3H - full blot images.pdf]

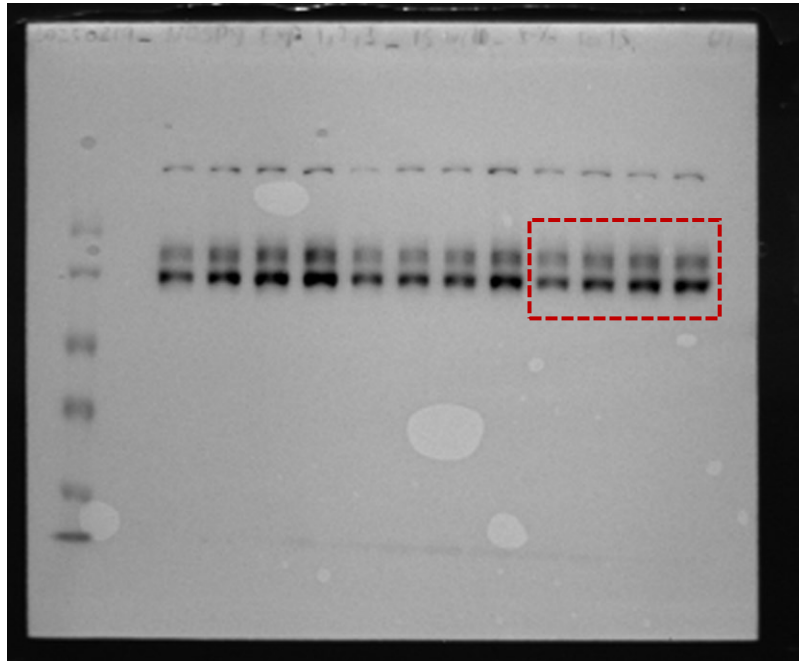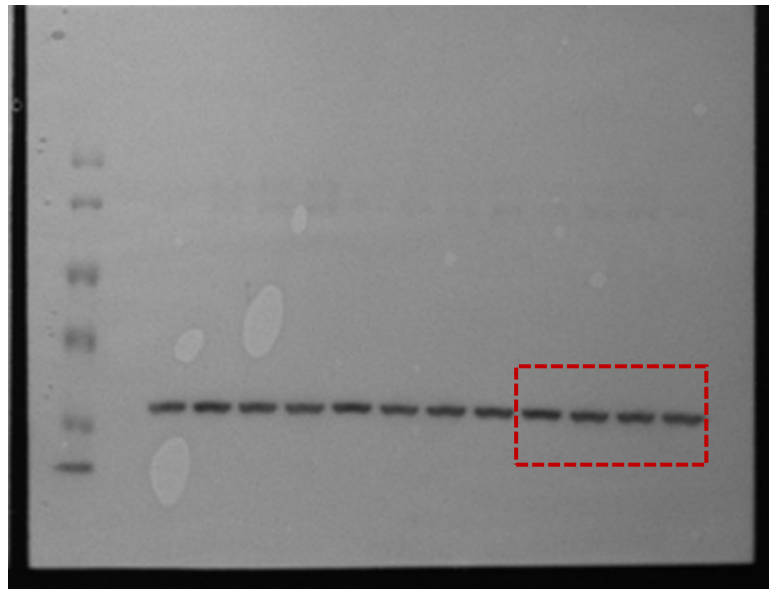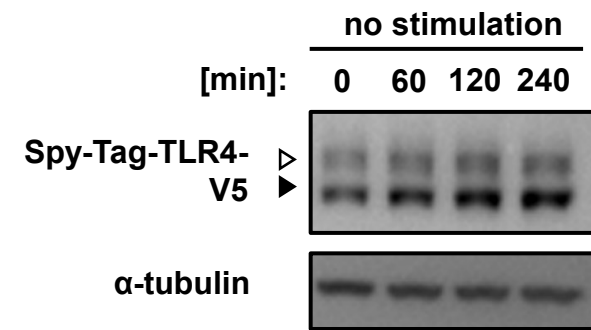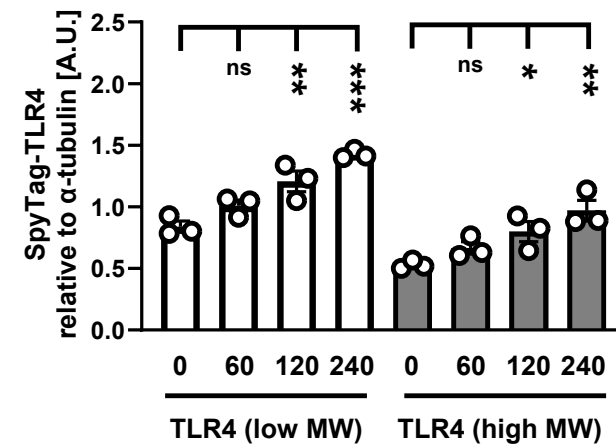

Supplement: Supplementary file 11 — Appendix Figures Source Data [file 44319_2025_444_MOESM11_ESM.zip › Appendix Figures/Appendix Figure S3/S3D/full WB images.pdf]

**Figure S8F:**

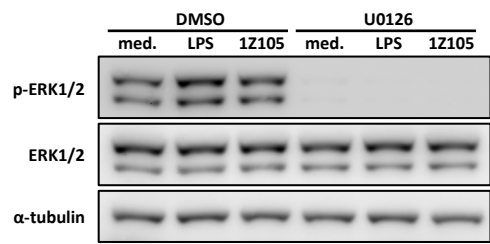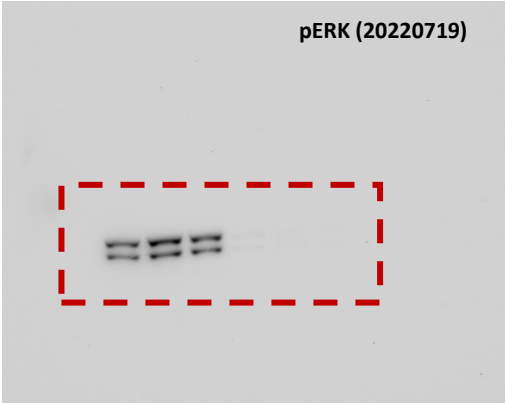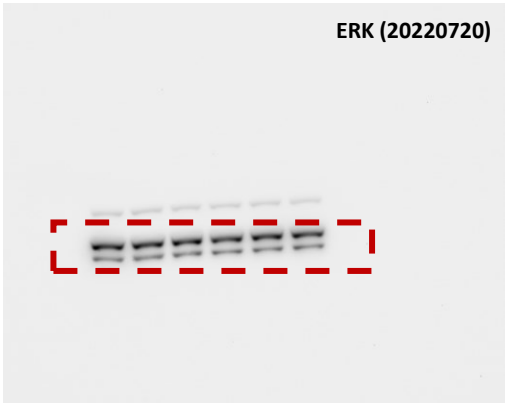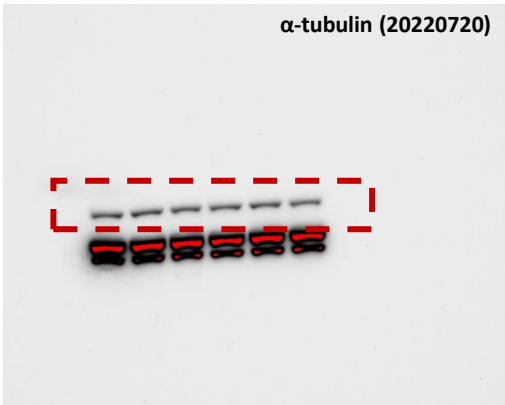

**Figure S8G:**

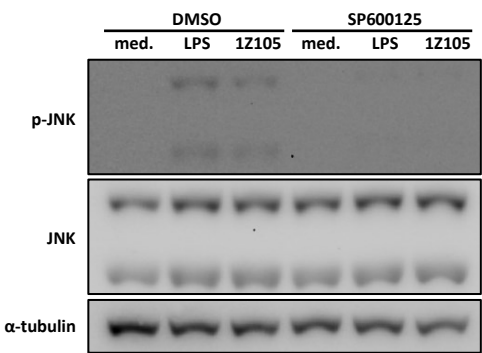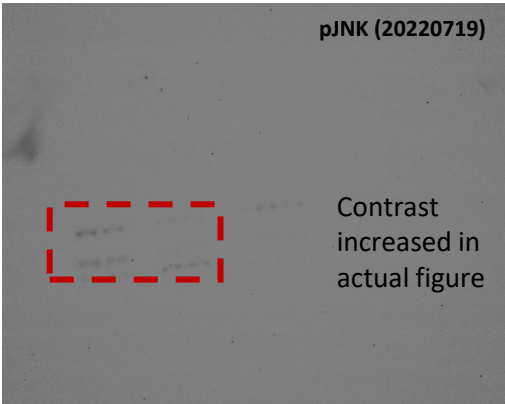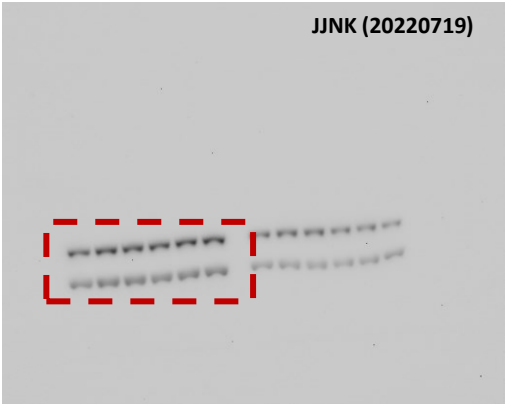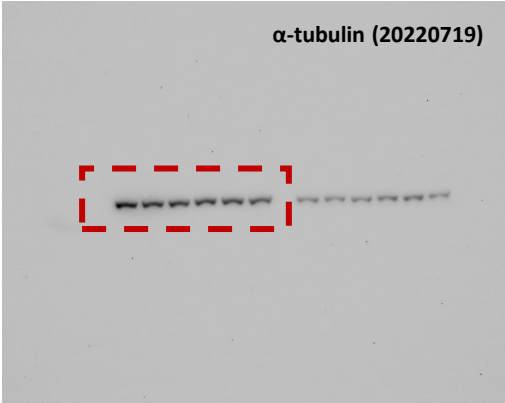

Supplement: Supplementary file 11 — Appendix Figures Source Data [file 44319_2025_444_MOESM11_ESM.zip › Appendix Figures/Appendix Figure S8/S8FG/S8FG - full blot images.pdf]
