# Supplementary material for: TLR4 endocytosis and endosomal TLR4 signaling are distinct and independent outcomes of TLR4 activation
Source: EMBO Rep. 2025 Apr 9;26(10):2740–66. doi: 10.1038/s44319-025-00444-2 (PMC12116916; doi:10.1038/s44319-025-00444-2)

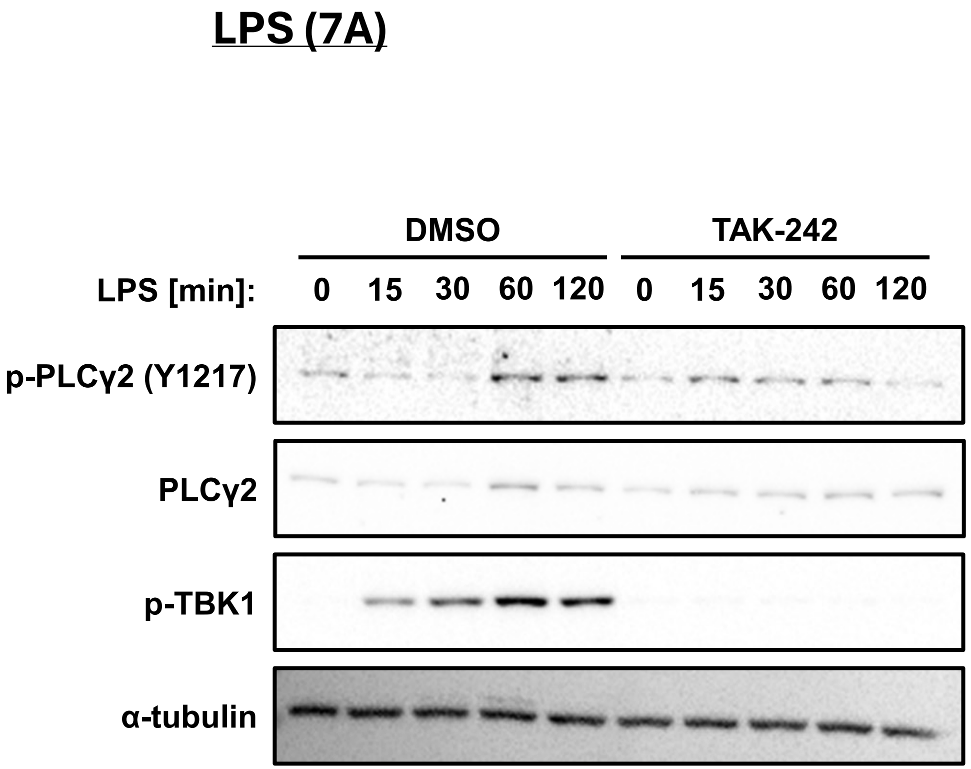
LPS (7A)
DMSO
TAK-242
LPS [min]: 0 15 30 60 120 0 15 30 60 120
p-PLCγ2 (Y1217)
PLCγ2
p-TBK1
α-tubulin

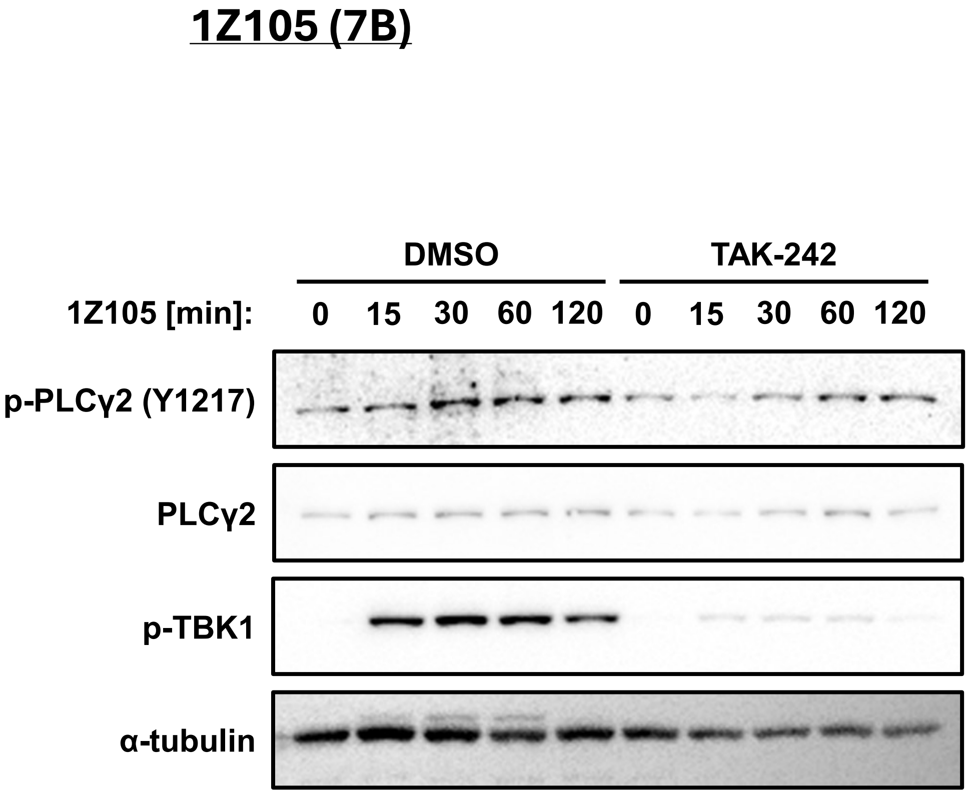
1Z105 (7B)
DMSO
TAK-242
1Z105 [min]: 0 15 30 60 120 0 15 30 60 120
p-PLCγ2 (Y1217)
PLCγ2
p-TBK1
α-tubulin

LPS (7A)

p-PLCγ2 (Y1217)

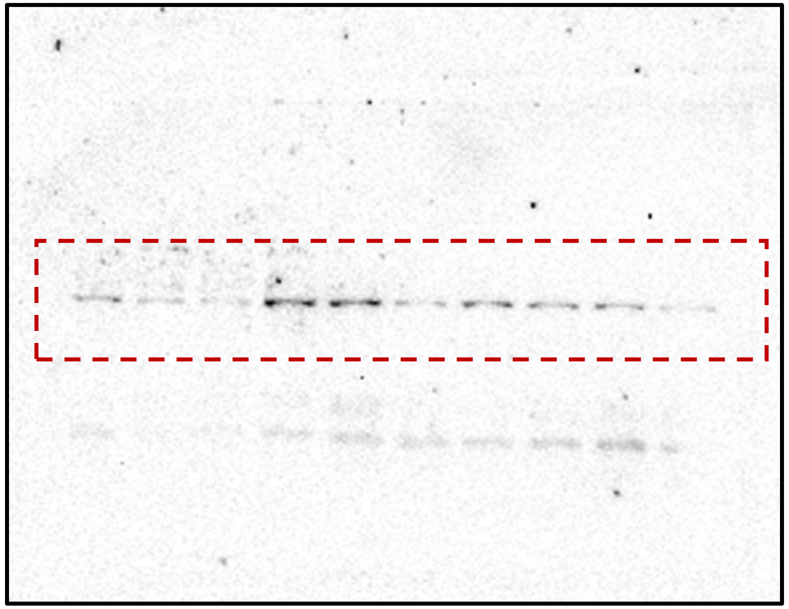

PLCγ2

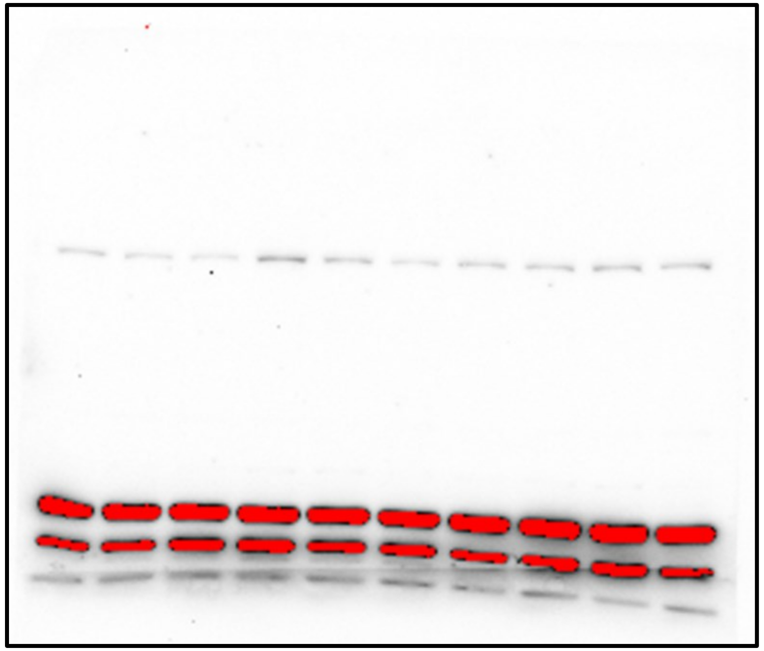

tubulin

Separate blot run for total PLC

α-tubulin

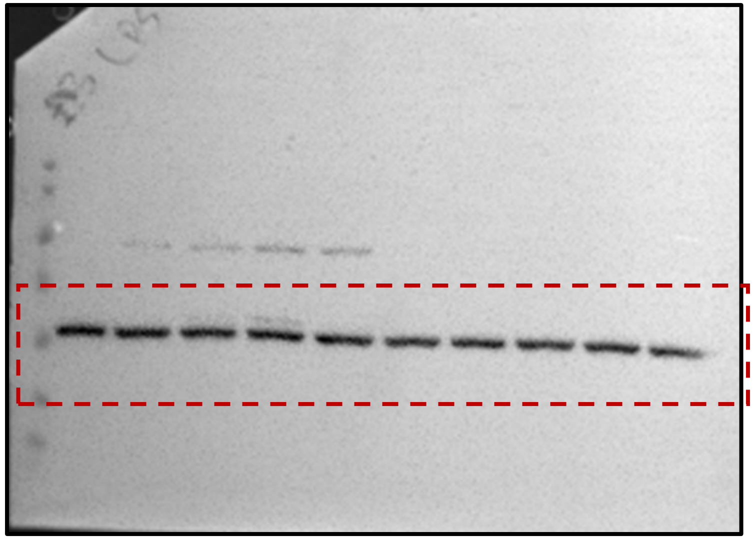

p-TBK1

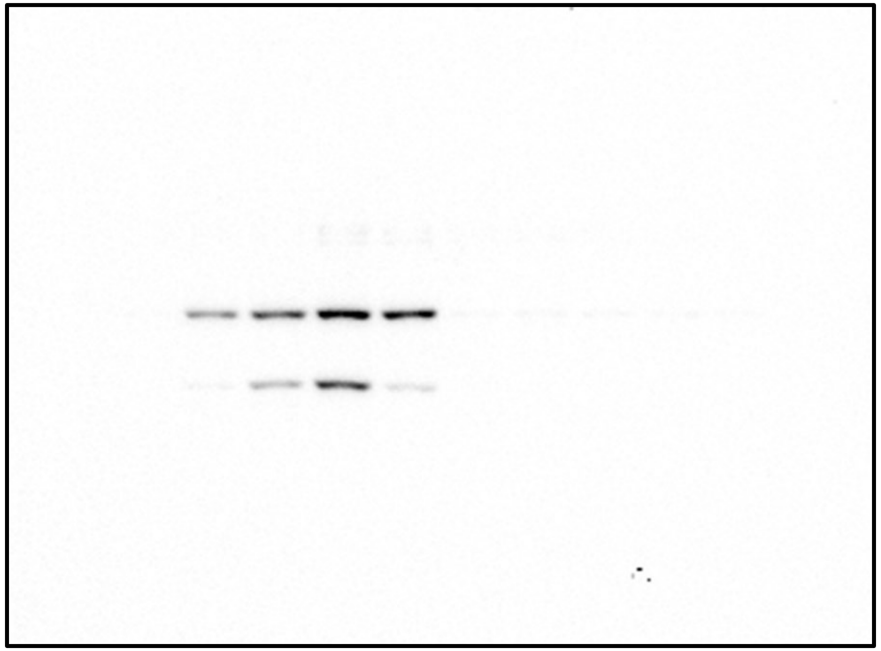

1Z105 (7B)

p-PLCγ2 (Y1217)

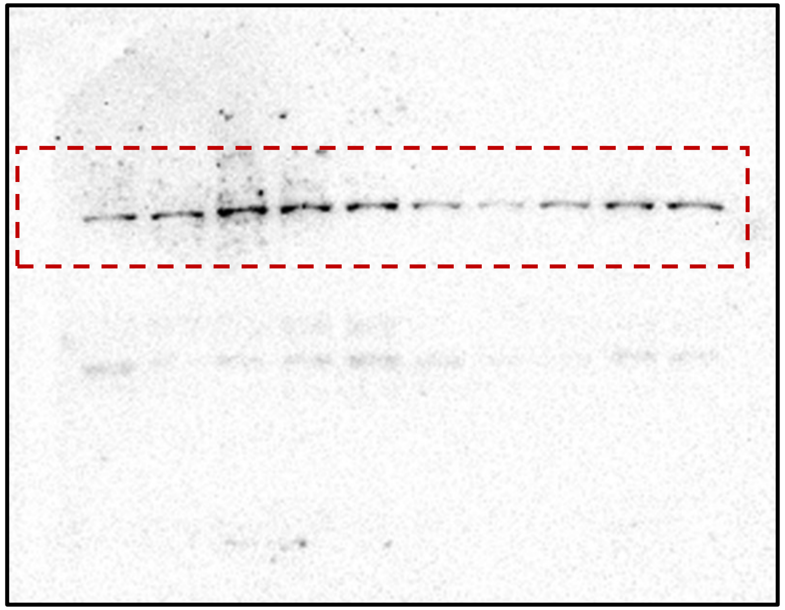

PLCγ2

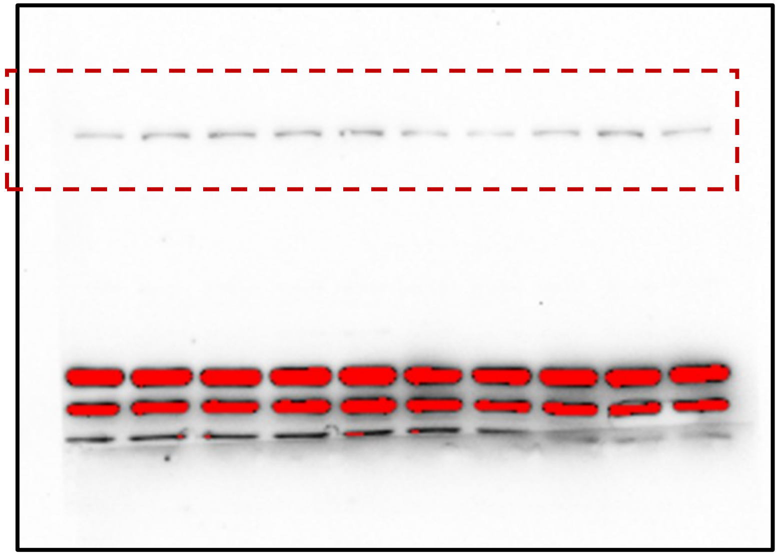

tubulin

Separate blot run for total PLC

α-tubulin

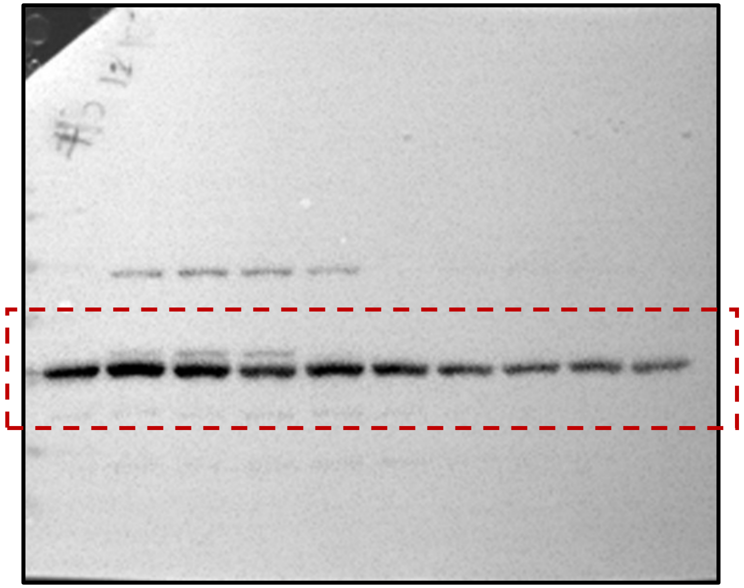

p-TBK1

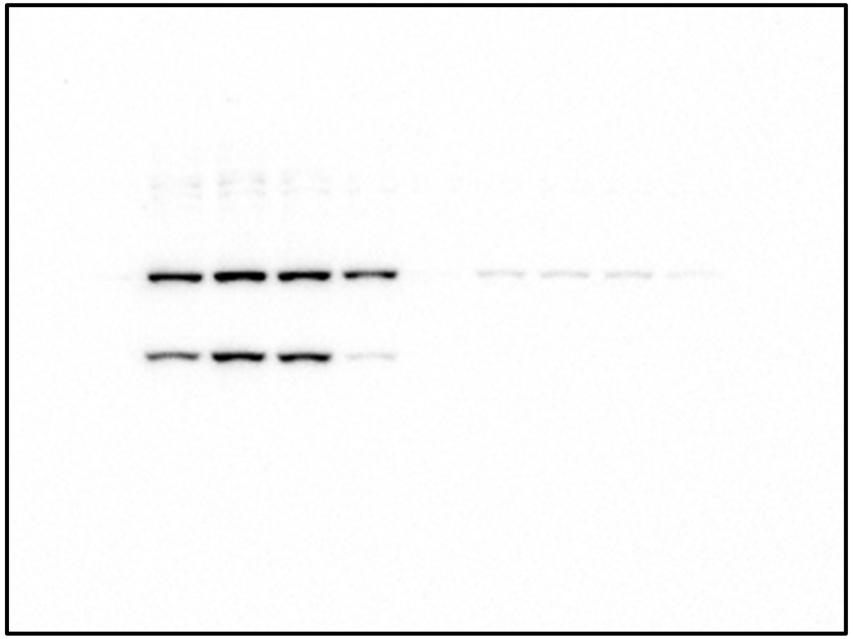

Supplement: Supplementary file 9 — Source data Fig. 7 [file 44319_2025_444_MOESM9_ESM.zip › Figure 7/7BC/7BC -full blot images.pdf]
