# Supplementary material for: TLR4 endocytosis and endosomal TLR4 signaling are distinct and independent outcomes of TLR4 activation
Source: EMBO Rep. 2025 Apr 9;26(10):2740–66. doi: 10.1038/s44319-025-00444-2 (PMC12116916; doi:10.1038/s44319-025-00444-2)

WT1 (mcs)

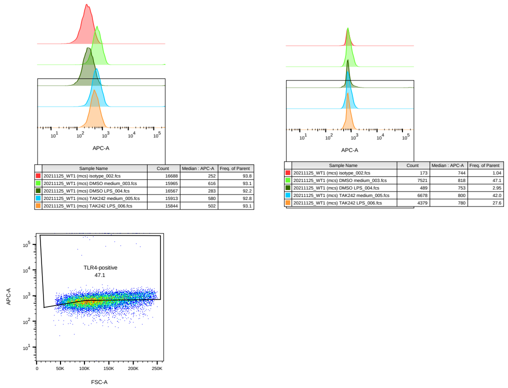

KO1 (mcs)

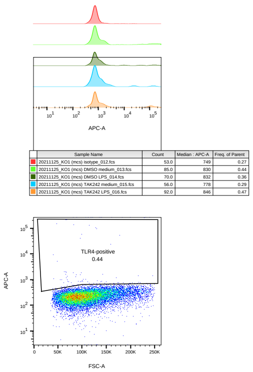

KO1 (WT)

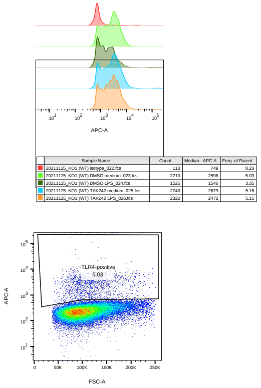

KO1 (C745A)

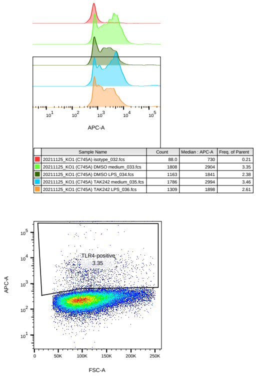

KO1 (C745S)

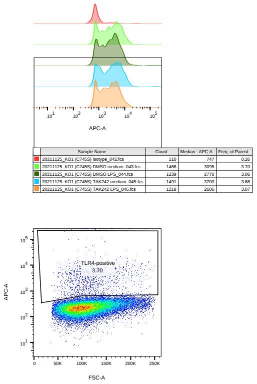

WT2 (mcs)

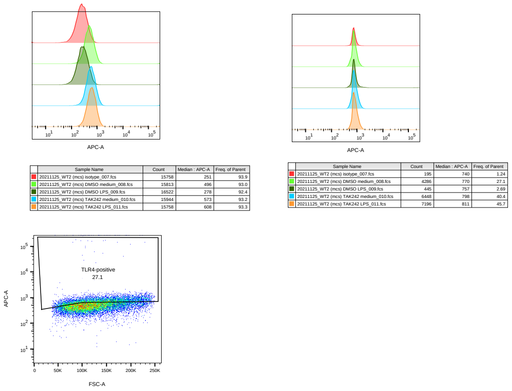

KO2 (mcs)

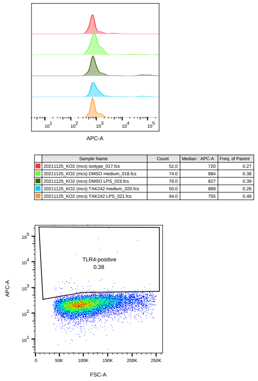

KO2 (WT)

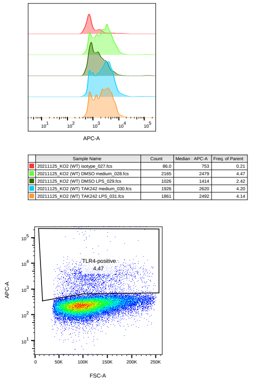

KO2 (C745A)

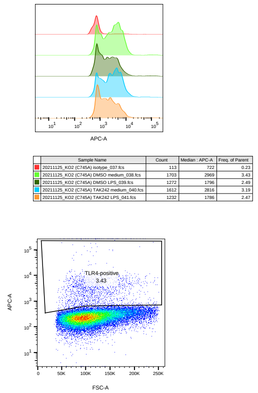

KO2 (C745S)

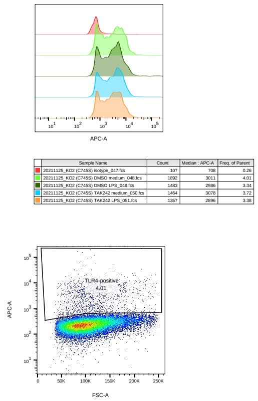

Supplement: Supplementary file 11 — Appendix Figures Source Data [file 27-Nov-2021-Layout.pdf › Appendix Figures/Appendix Figure S6/S6BC/27-Nov-2021-Layout.pdf]
