# Supplementary material for: TLR4 endocytosis and endosomal TLR4 signaling are distinct and independent outcomes of TLR4 activation
Source: EMBO Rep. 2025 Apr 9;26(10):2740–66. doi: 10.1038/s44319-025-00444-2 (PMC12116916; doi:10.1038/s44319-025-00444-2)
Supplement: Supplementary file 12 — Expanded View Figures [file 44319_2025_444_MOESM12_ESM.pdf]

Expanded View Figures

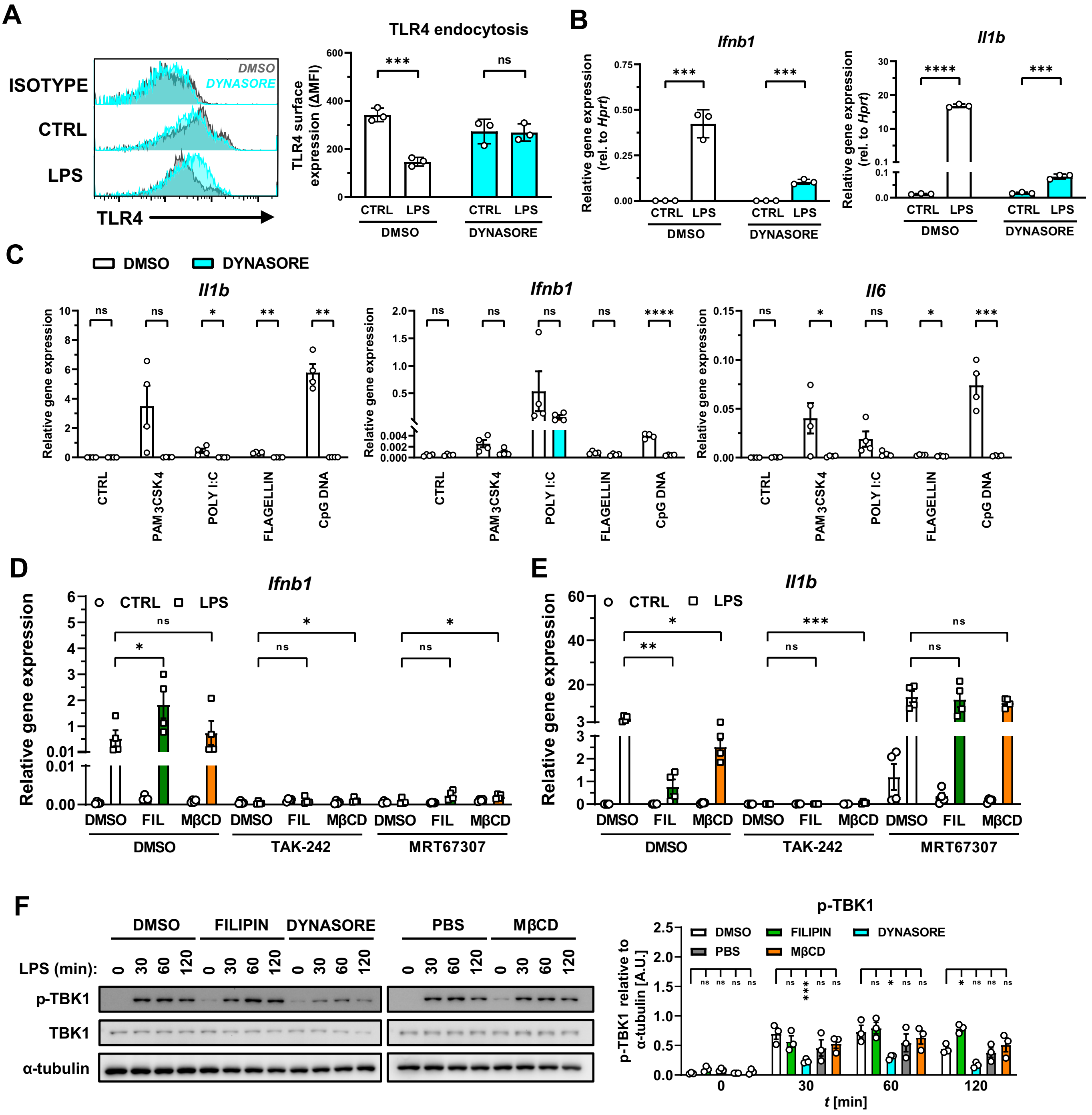

Figure EV1. Impact of dynasore and lipid raft inhibitors on TLR-mediated macrophage activation.

(**A**) Flow cytometric analysis of TLR4 surface expression in WT BMM treated for 60 min with DMSO or dynasore (80 μM), followed by stimulation with LPS (100 EU/mL) or left unstimulated (CTRL) for 120 min. (**B**) qRT-PCR analysis of *Ifnb1* and *Il1b* expression in WT BMM treated for 60 min with DMSO or dynasore (80 μM), followed by stimulation with LPS (100 EU/mL) for 90 min. (**C**) qRT-PCR analysis of *Il1b*, *Ifnb1* and *Il6* mRNA expression in WT BMM treated for 60 min with DMSO or dynasore (80 μM), followed by stimulation with Pam_3CSK_4 (10 ng/mL), poly I:C (10 μg/mL), flagellin (0.5 μg/mL) or CpG DNA (1 μM), or left unstimulated (CTRL), for 90 min. (**D**, **E**) Impact of TAK-242 (1 μM) or MRT67307 (5 μM) on LPS-induced (**D**) *Ifnb1* and (**E**) *Il1b* expression (90 min) in WT BMM treated with filipin (5 μM), MβCD (10 mM), or DMSO as control. (**F**) Time-course of LPS-induced TBK1 phosphorylation in WT BMM treated with filipin, MβCD, dynasore or DMSO as solvent control. Data information: Flow cytometry histograms and immunoblot images depict 1 representative of $n = 3$ biological replicates generated in independent experiments. Bar plots are mean ± SEM of $n = 3$–4 biological replicates generated in independent experiments indicated as data points. Unpaired two-tailed *t* test performed in (**A**–**C**); ordinary two-way ANOVA with Dunnett's multiple comparison test utilized in (**D**–**F**); $^{*}P < 0.05$; $^{**}P < 0.01$; $^{***}P < 0.001$, $^{****}P < 0.0001$, ns = not significant. Source data are available online for this figure.

© The Author(s)

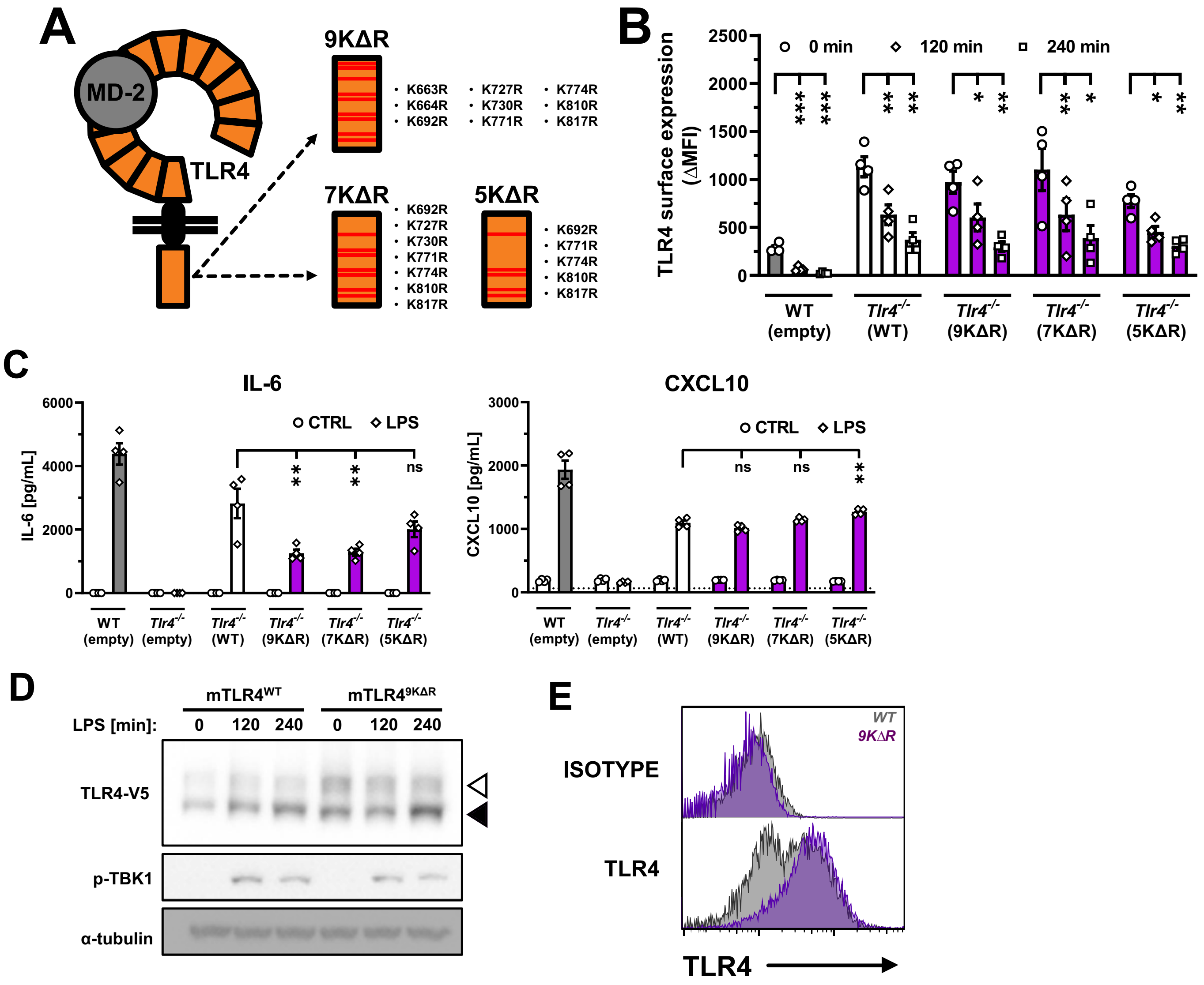

Figure EV2. Lysine residues in the TLR4 TIR domain were not required for LPS-induced TLR4 endocytosis.

(A–C) *Tlr4*$^{-/-}$ BMMs retrovirally reconstituted with (A) mTLR4^{WT}, mTLR4$^{9K\Delta R}$, mTLR4$^{7K\Delta R}$, mTLR4$^{5K\Delta R}$ or empty vector were stimulated with LPS (100 EU/mL); (B) TLR4 surface expression assessed at 120 and 240 min post-stimulation and (C) IL-6 and CXCL10 production assessed at 24 h post-stimulation. (D, E) RAW^{TLR4ko} cells were stably transfected with V5-tagged constructs of mTLR4^{WT} or mTLR4$^{9K\Delta R}$. (D) Total cellular expression of TLR4-V5 during LPS stimulation (100 EU/mL, 0, 120, 1240 min) was assessed via immunoblot. Phospho-TBK1 served as control for cellular activation; a-tubulin served as loading control. Black arrow head indicates low glycosylated intracellular TLR4, white arrow head indicates highly-glycosylated cell surface-expressed TLR4. (E) TLR4 surface expression was assessed in resting cells via flow cytometry. Data information: Immunoblot images depict 1 representative of $n = 3$ biological replicates generated in independent experiments. Flow cytometry histogram represents cells post sorting. Bar plots are mean ± SEM of $n = 3$–4 biological replicates generated in independent experiments indicated as data points. RM two-way ANOVA with Dunnett's multiple comparisons test utilized in (B). Ordinary one-way ANOVA with Dunnett's multiple comparisons test utilized in (C). *$P < 0.05$; **$P < 0.01$; ***$P < 0.001$, ****$P < 0.0001$, ns = not significant. Source data are available online for this figure.

 © The Author(s)

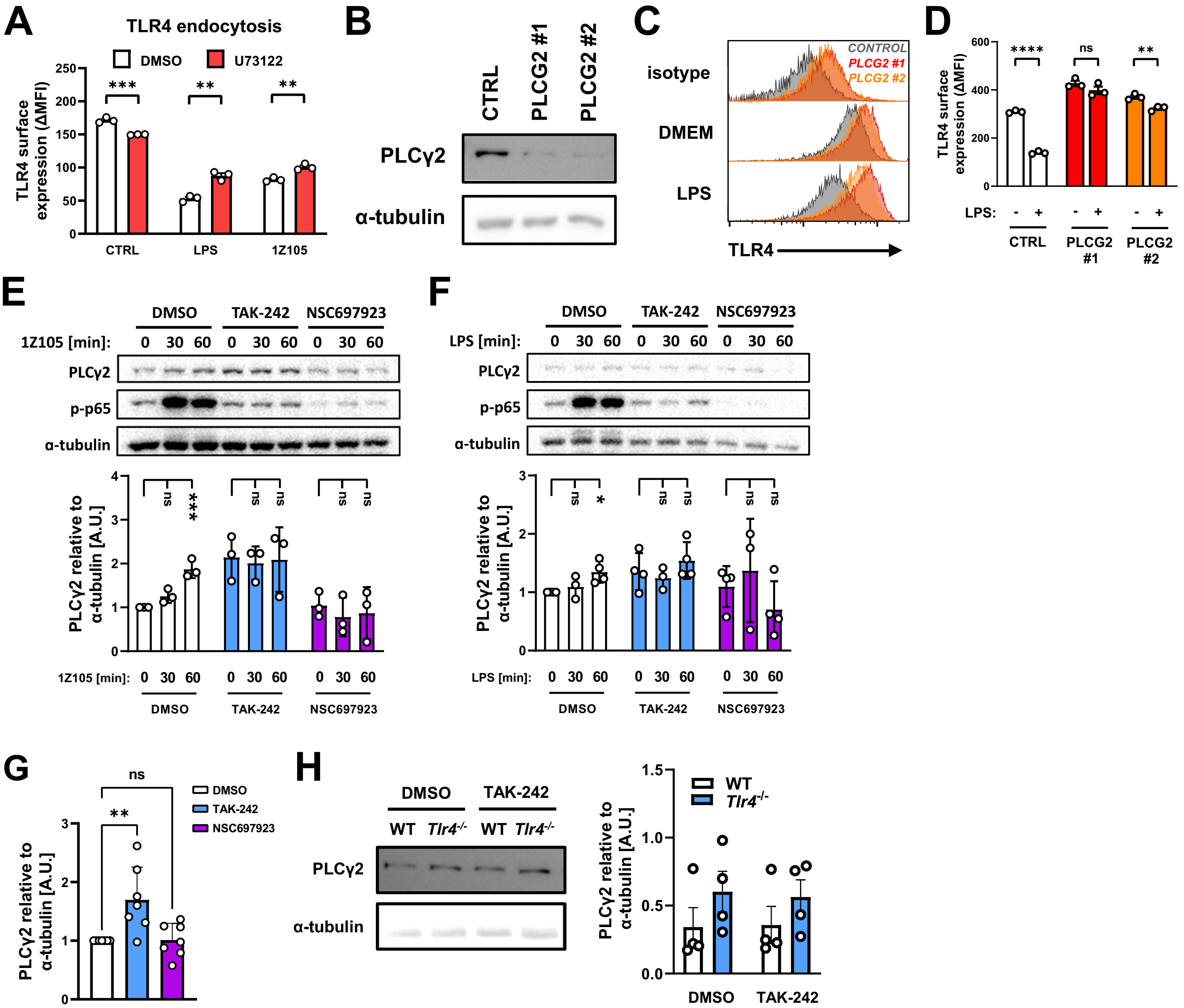

Figure EV3. PLCγ2 regulates TLR4 endocytosis.

(**A**) Impact of PLC inhibitor (U73122, 10 μM) on LPS- (100 EU/mL) and 1Z105-induced (10 μM) TLR4 endocytosis (120 min) in WT BMM. (**B**–**D**) CRISPR/Cas9-mediated knockdown of PLCγ2 expression in WT BMM (**B**) was verified by immunoblot analysis, and (**C**, **D**) shown to impair LPS-induced (100 EU/mL, 120 min) TLR4 endocytosis as assessed by flow cytometry. (**E**, **F**) Immunoblot analysis of the impact of TAK-242 (1 μM) or NSC694923 (10 μM) on total PLCγ2, phosphorylated NF-κB p65 and α-tubulin in WT BMM upon stimulation with (**C**) 1Z105 (10 μM) or (**D**) LPS (100 EU/mL) for the indicated times. (**G**) Comparison of PLCγ2 expression in unstimulated cells presented in (**E**, **F**). (**H**) Immunoblot analysis of PLCγ2 cellular expression in WT and *Tlr4*$^{-/-}$ BMM treated with DMSO or TAK-242 (1 μM) for 120 min. Data information: Flow cytometry histograms and immunoblot images depict 1 representative of $n = 3$–4 biological replicates generated in independent experiments. Bar plots are mean ± SEM of $n = 3$–7 biological replicates generated in independent experiments indicated as data points. Unpaired two-tailed t test utilized in (**A**, **D**). Ordinary one-way ANOVA with Dunnett's multiple comparisons test utilized in (**E**–**G**). *$P < 0.05$; **$P < 0.01$; ***$P < 0.001$, ****$P < 0.0001$, ns = not significant. Source data are available online for this figure.
